# The Response of *Cupriavidus metallidurans* CH34 to Cadmium Involves Inhibition of the Initiation of Biofilm Formation, Decrease in Intracellular c-di-GMP Levels, and a Novel Metal Regulated Phosphodiesterase

**DOI:** 10.3389/fmicb.2019.01499

**Published:** 2019-07-09

**Authors:** Pablo Alviz-Gazitua, Sebastián Fuentes-Alburquenque, Luis A. Rojas, Raymond J. Turner, Nicolas Guiliani, Michael Seeger

**Affiliations:** ^1^Laboratorio de Comunicación Bacteriana, Departamento de Biología, Facultad de Ciencias, Universidad de Chile, Santiago, Chile; ^2^Laboratorio de Microbiología Molecular y Biotecnología Ambiental, Departamento de Química and Centro de Biotecnología, Universidad Técnica Federico Santa María, Valparaíso, Chile; ^3^Ph.D. Program of Microbiology, Facultad de Ciencias, Universidad de Chile, Santiago, Chile; ^4^Microbial Ecology of Extreme Systems Laboratory, Facultad de Ciencias Biológicas, Pontificia Universidad Católica de Chile, Santiago, Chile; ^5^Department of Chemistry, Universidad Católica del Norte, Antofagasta, Chile; ^6^Biofilm Research Group, Department of Biological Sciences, University of Calgary, Calgary, AB, Canada

**Keywords:** c-di-GMP, *Cupriavidus metallidurans*, cadmium, phosphodiesterase, biofilm, *urf2* gene, *mer* gene, PleD

## Abstract

Cadmium is a highly toxic heavy metal for biological systems. *Cupriavidus metallidurans* CH34 is a model strain to study heavy metal resistance and bioremediation as it is able to deal with high heavy metal concentrations. Biofilm formation by bacteria is mediated by the second messenger *bis*-(3′–5′)-cyclic dimeric guanosine monophosphate (c-di-GMP). The aim of this study was to characterize the response of *C. metallidurans* CH34 planktonic and biofilm cells to cadmium including their c-di-GMP regulatory pathway. Inhibition of the initiation of biofilm formation and EPS production by *C. metallidurans* CH34 correlates with increased concentration of cadmium. Planktonic and biofilm cells showed similar tolerance to cadmium. During exposure to cadmium an acute decrease of c-di-GMP levels in planktonic and biofilm cells was observed. Transcription analysis by RT-qPCR showed that cadmium exposure to planktonic and biofilm cells induced the expression of the *urf2* gene and the mercuric reductase encoding *merA* gene, which belong to the Tn*501*/Tn*21 mer* operon. After exposure to cadmium, the *cadA* gene involved in cadmium resistance was equally upregulated in both lifestyles. Bioinformatic analysis and complementation assays indicated that the protein encoded by the *urf2* gene is a functional phosphodiesterase (PDE) involved in the c-di-GMP metabolism. We propose to rename the *urf2* gene as *mrp* gene for metal regulated PDE. An increase of the second messenger c-di-GMP content by the heterologous expression of the constitutively active diguanylate cyclase PleD correlated with an increase in biofilm formation and cadmium susceptibility. These results indicate that the response to cadmium in *C. metallidurans* CH34 inhibits the initiation of biofilm lifestyle and involves a decrease in c-di-GMP levels and a novel metal regulated PDE.

## Introduction

Cadmium is a highly toxic heavy metal for biological systems. Cadmium induces indirectly oxidative stress, disturbs manganese acquisition, and depletes the cellular reduced thiol pool (Vallee and Ulmer, 1972; Nies, 1999; Begg et al., 2015). This heavy metal is also carcinogenic (World Health Organization [WHO], 2011). Global cadmium production has risen >1,000-fold in the last century due to its incorporation into diverse manufacture applications (Begg et al., 2015). The Word Health Organization included cadmium in the top 10 of the “chemicals of major public health concern” (World Health Organization [WHO], 2011).

The remediation of heavy metals in polluted waters and soils is a major challenge for sustainable development (Orellana et al., 2018). Microorganisms are used to immobilize and/or transform heavy metals into less toxic forms (Rojas et al., 2011; Mosa et al., 2016; Orellana et al., 2018). Heavy metal bioremediation is commonly carried out in fixed bed bioreactors wherein the formation of attached multicellular communities – i.e., biofilm – is crucial. Biofilm cells produce extracellular polymeric substances (EPS) such as polysaccharides, lipids, proteins, and extracellular DNA, providing different functional groups that may bind heavy metal ions via passive biosorption processes that immobilize heavy metals and protect cells (Harrison et al., 2007). Biofilms are also involved in bioreduction processes through electrically conductive components (Harrison et al., 2004b; Chen et al., 2018; Espinoza-Tofalos et al., 2018). Biofilms have been described as an advantageous lifestyle promoted by stress, toxic compounds, or heavy metals, which allow the selection of resistant cells (Harrison et al., 2007). However, in contrast to the bacterial response to antibiotics, planktonic and biofilm cells are in most cases almost equally susceptible to heavy metal oxyanions and cations (Harrison et al., 2004b; Gugala et al., 2017). Consequently, the minimum biofilm-eradication concentration (MBEC) is often not higher than the minimum bactericidal concentration (MBC) observed under planktonic growth conditions. Specific bacterial biofilms are even more susceptible to some heavy metal oxyanions and cations than planktonic cells (Harrison et al., 2004a). Metal tolerance in bacterial biofilms is dependent on the bacterial strain, the metal, and the culture medium (Harrison et al., 2004a, 2005; Gugala et al., 2017).

Biofilm formation in response to environmental conditions is mediated by the second messenger *bis*-(3′–5′)-cyclic dimeric guanosine monophosphate (c-di-GMP) (Jenal et al., 2017). Intracellular pools of c-di-GMP seem to be dynamically regulated by diguanylate cyclases (DGCs) and c-di-GMP-specific phosphodiesterases (PDEs) that catalyze the formation and the degradation of c-di-GMP, respectively. DGC activity depends on the characteristic amino acid motif GGDEF (Wassmann et al., 2007), whereas PDE activity is mediated by the two amino acid motifs EAL and HD-GYP (Tchigvintsev et al., 2010; Lovering et al., 2011). In addition to the catalytic motifs, DGCs and PDEs contain accessory motifs, which are responsible for the regulation of their activity under different conditions. Most DGCs and PDEs act at local manner and do not impact the cellular c-di-GMP levels (Sarenko et al., 2017; Dahlstrom et al., 2018). At high concentrations, c-di-GMP interacts with a broad spectrum of c-di-GMP binding effectors including riboswitches and a wide diversity of proteins. The effectors include GGDEF/EAL domain proteins (Duerig et al., 2009; Jain et al., 2017) and PilZ domain proteins (Habazettl et al., 2011). The c-di-GMP binding with specific effectors may trigger a reduction of cell motility and an increase of EPS matrix synthesis, which promote biofilm formation (Duerig et al., 2009; Römling et al., 2013; Hengge et al., 2016; Jain et al., 2017). The c-di-GMP signaling system is more complex, since flagella play roles in biofilm formation and matrix synthesis during stationary phase in liquid culture (Hengge et al., 2016). However, the link between the c-di-GMP signaling pathway, bacterial lifestyle, and heavy metal tolerance has not yet been explored.

*Cupriavidus metallidurans* CH34 is a model heavy metal-resistant strain that can tolerate millimolar concentrations of heavy metal concentrations. Strain CH34 has been used for bioremediation, recovery, and reduction of heavy metals (Mergeay et al., 1985; Collard et al., 1994; Janssen et al., 2010). *C. metallidurans* CH34 harbors numerous heavy metal resistance determinants involved in diverse mechanisms, including complexation, efflux systems, reduction, and reductive precipitation, which enable cell detoxification and survival (Nies, 2016; Maes et al., 2017; Montero-Silva et al., 2018). A high number of CH34 gene products confer tolerance to high concentrations of heavy metals, including the CzcCBA, CzcD, CzcP, and CadA proteins that are involved in cadmium resistance (Nies, 2016). Nevertheless, molecular mechanisms driving the bacterial lifestyle in the presence of heavy metals remain unexplored. The aim of this study was to characterize the response to cadmium of *C. metallidurans* CH34 planktonic and biofilm cells including their c-di-GMP regulatory pathway.

## Materials and Methods

### Organisms, Plasmids, and Growth Conditions

Bacterial strains and plasmids used in this study are listed in Table 1. *C. metallidurans* strains were cultured in low phosphate Tris-buffered mineral salts (LPTMS) broth [Tris 6.06 g L^-1^, NaCl 4.68 g L^-1^, NH**_4_**Cl 1.07 g L^-1^, KCl 1.49 g L^-1^, Na**_2_**SO_4_⋅10H_2_O 0.98 g L^-1^, MgCl_2_⋅6H_2_O 0.2 g L^-1^, CaCl**_2_**⋅2H_2_0 0.03 g L^-1^, Na**_2_**HPO**_4_** 0.085 g L^-1^, Fe(III)(NH**_4_**) citrate 0.005 g L^-1^, succinate 0.3%, and 1 mL L^-1^ of SL7 trace solution of Biebl and Pfennig] (Mergeay et al., 1985). The culture medium was adjusted to pH 7.0. *Escherichia coli* and *Pseudomonas aeruginosa* strains were cultured in LB broth (yeast extract 5 g L^-1^, peptone 10 g L^-1^, NaCl 5 g L^-1^). In this study, plasmids pJB3Tc19 (Blatny et al., 1997) and pJB*pleD^∗^* (Pérez-Mendoza et al., 2014) were used. The PleD^∗^ protein is a DGC from *Caulobacter crescentus* that contains four point mutations and retains its activity independently of phosphorylation input by a cognate kinase (Paul et al., 2004).

**Table 1:** Plasmids and strains used in this study.

Plasmids/strains	Description	References
pJB3Tc19	Ap^r^, Tc^r^; expression vector	Blatny et al., 1997
pJB*urf2.2*	Ap^r^, Tc^r^; pJBTc19 derivate bearing a 1423 bp *Xba*I/*Eco*RI fragment containing *RMET_RS31035*	This study
pJB*pleD*^∗^	Ap^r^, Tc^r^; pJB3Tc19 derivate bearing a 1423 bp *Xba*I/*Eco*RI fragment containing *pleD^∗^*	Pérez-Mendoza et al., 2014
*C. metallidurans* CH34	Heavy metal multiresistant strain	Mergeay et al., 1985
CH34 pJB3Tc19	CH34 strain transformed with an empty vector	This study
CH34 pJB*urf2.2*	CH34 strain transformed with pJB*urf2.2* construct	This study
CH34 pJB*pleD^∗^*	CH34 strain transformed with pJB*pleD^∗^* construct	This study
*P. aeruginosa* PAO1	Opportunistic pathogen	Rao et al., 2008
Δ*rocR* pJB3Tc19	*roc*R null mutant PAO1 strain transformed with pJB3Tc19	This study
Δ*rocR* pJB*urf2.2*	*roc*R null mutant PAO1 strain transformed with pJB*urf2.2*	This study

### Biofilm Formation Assay

*Cupriavidus metallidurans* CH34 cell attachment in the absence or presence of cadmium was assessed by an adapted protocol developed to monitor initial stages of biofilm formation (O’Toole and Kolter, 1998; Merritt et al., 2007). Briefly, a stationary culture in LPTMS broth at 30°C was centrifuged and the cells were suspended in fresh LPTMS broth to a turbidity at 600 nm of 1.0. The culture was distributed by placing 200 μL per well into a 96-well microtiter plate. A concentrated cadmium solution was added to reach different concentrations. After 15 h of incubation at 30°C in static condition, planktonic cells were removed. Wells were washed with sterile distilled water and the biofilm adhered to the bottom was stained with crystal violet (200 μL per well, 0.1% solution for 15 min). Plate was air-dried for 16 h and the stained biomass was suspended in 200 μL of acetic acid 30%. Absorbance of this suspension was measured either at 550 or 595 nm. This value was normalized by the turbidity_600nm_ of the planktonic fraction removed previously. Viability of each planktonic cell fraction was quantified by serial dilution and plating in triplicate on tryptic soy agar (pancreatic digest of casein 15 g L^-1^, peptic digest of soybean meal 5 g L^-1^, sodium chloride 5 g L^-1^, and agar 15 g L^-1^). Colony forming units (CFUs) were counted on plates.

### Qualitative Assay for Extracellular Polymeric Substances

Levels of EPS synthesis by *C. metallidurans* CH34 macrocolonies were assessed in salt-free LB agar in presence of Congo Red 50 μg mL^-1^ and Coomassie blue 10 μg mL^-1^ (Romero et al., 2010). Plates were incubated for 5 days at room temperature and visualized with an Olympus MVX10 lens. The red staining intensity of the macrocolony correlates to its EPS content.

### Analysis of Biofilm and Planktonic Lifestyles of *C. metallidurans* CH34

For comparisons between biofilm and planktonic lifestyles, two aliquots of an LPTMS broth culture (10^8^ cells mL^-1^) were taken in parallel. For biofilm growth, 1 mL was vacuum filtered through a 0.2-μm polycarbonate filter (Whatman) and the filter was placed on solid LPTMS (0.8% agar) with the cell filtrate facing upward. The second 1 mL aliquot was inoculated into 9 mL of fresh LPTMS broth for planktonic growth. The cultures were incubated at 30°C. The biofilm was incubated without shaking, whereas the planktonic culture was incubated at 150 rpm. This procedure synchronized cultures of both lifestyles starting from the same growth phase and biomass. After 13 h at 30°C, cultures were exposed to 2 mM cadmium (Supplementary Figure S1), either by translating the filter to a new LPTMS agar with cadmium (biofilm cells) or by directly adding a cadmium concentrated solution (planktonic cells). After 45 min incubation in presence of cadmium, cells were processed for c-di-GMP determination and qRT-PCR.

### Extraction and Quantification of c-di-GMP

After 45 min incubation in presence or absence of cadmium, 9 of the 10 mL of planktonic culture were collected, leaving 1 mL for total protein content determination. To discard sample processing bias, biofilm cells were suspended in a mixture of 10 mL of LPTMS, separating a 1 mL aliquot for total protein content determination. Cells in suspension were collected by centrifugation at 13,000 × *g* for 10 min at 4°C. Pellets were suspended in 300 μL of extraction solution (acetonitrile:methanol:water, 2:2:1) in 2 mL microtube vials and incubated 10 min at 95°C followed by cooling on ice. Cell lysates were centrifuged at 13,000 × *g* for 10 min at 4°C and supernatants were transferred into new microtube vials. Extractions were repeated twice omitting the heat incubation step. Combined supernatants of three extraction steps (about 700 μL) were stored at -20°C overnight. Remaining cellular debris was removed by centrifugation at 13,000 × *g* for 10 min at 4°C. Finally, supernatants were evaporated to dryness in Speed-Vac at room temperature. Nucleotide extraction from planktonic cells of *P. aeruginosa* strains followed the same procedure. Quantification of c-di-GMP was achieved by temperature-controlled liquid chromatography (using a C18 RP column) coupled to tandem mass spectrometer (LC–MS/MS) equipped with an electro spray ionization (ESI) source (positive ionization mode) at BIOLOG Life-Sciences Institute (Bremen, Germany) based on the method reported previously (Spangler et al., 2010).

### Susceptibility Tests in Calgary Biofilm Device

*Cupriavidus metallidurans* cultures in the Calgary Biofilm Device (96-well format with lid containing pegs that insert into each well) were performed as reported (Harrison et al., 2010). Briefly, colonies grown on LPTMS agar during 48 h incubation at 30°C were suspended in NaCl 0.9% to reach a turbidity equivalent to McFarland standard 1.0 (Thermo R20421). The suspension was diluted (15-fold) in LPTMS broth, loaded into the Calgary Biofilm Device (Innovotech) and incubated for 24 h at 150 rpm and 30°C. Different cadmium concentrations were titrated across the microtiter plates. After incubation, the peg lids were removed thus separating the biofilms adhered to the lid pegs from the planktonic fraction in the well. Cadmium susceptibility was determined by CFU counting in tryptic soy agar after 24 h of cadmium challenge incubations. MBC and MBEC were defined as the point where no growth was observed on the plate (Workentine et al., 2008).

### Determination of Minimal Inhibitory Concentration (MIC)

Minimal inhibitory concentration was defined as the cadmium concentration where no increase of turbidity at 600 nm was observed in microtiter plate cultures after 96 h incubation at 150 rpm and 30°C (Monsieurs et al., 2011).

### Functional Prediction of Genes Encoding DGCs, PDEs, and PilZ Domain Effectors in the *C. metallidurans* CH34 Genome

A list of genes coding for DGCs and PDEs in the *C. metallidurans* CH34 genome (Supplementary Table S1) was retrieved from the NCBI website http://www.ncbi.nlm.nih.gov/Complete_Genomes/c-di-GMP.html, according to NCBI annotations. GGDEF, EAL, and HD-GYP domain proteins coding genes were analyzed. The catalytic functionality of each protein was evaluated by analyzing the conservation of the active amino acid residues in domain sequence motifs residues Asp327, Lys332, Asn335, Asp344, Asp370, Lys442, and Arg446 for GGDEF; residues Glu523, Arg527, Glu546, Asn584, Glu616, Asp646, Asp647, Glu703, and Gln723 for EAL; and residues His183, Asp184, His212, His237, Glu(His)238, Asn(Glu)265, and Asn(Arg)269 for HD-GYP, using as reference functional domains of PleD (GGDEF) from *Caulobacter crescentus* (Wassmann et al., 2007), TBD_1265 (EAL) from *Thiobacillus denitrificans* (Tchigvintsev et al., 2010), and Bd1817 (HD-GYP) from *Bdellovibrio bacteriovorus* (Lovering et al., 2011), respectively. Motif conservation in GGDEF, EAL, and HD-GYP domains was determined by multiple sequence alignment using Clustal W (Thompson et al., 1994).

The c-di-GMP binding capability of the PilZ domains of *C. metallidurans* CH34 was predicted based on the conservation of the “c-di-GMP switch” domain (RxxxR and D/NzSxxG), which undergoes the conformational change that wraps around the c-di-GMP molecule (Duerig et al., 2009). The PilZ domain of PA4608 protein from *P. aeruginosa* was used as the active form model (Römling et al., 2013), whereas the PilZ domain of protein XC1028 from *Xanthomonas campestris* was used as the inactive form reference (Li et al., 2009). The search of c-di-GMP effectors related to *Burkholderia cenocepacia* Bcam1349 (Fazli et al., 2014) and *P. aeruginosa* PelD in the *C. metallidurans* CH34 genome was assessed by local alignment tools and pairwise sequence alignment. Motif conservation in PilZ domains was determined by global multiple sequence alignment using the MAFFT software that identifies rapidly homologous regions by the fast Fourier transform (Katoh et al., 2002). Motif conservation was visualized using Jalview software^1^ reported by Waterhouse et al. (2009). Orthology inference was achieved through sequence similarity searching using BlastP algorithm (Altschul et al., 1997) and confirmed by conservation of domains architectures obtained from NCBI’s CDART tool (Geer et al., 2002).

### RNA Extraction and Reverse Transcription

After incubation of cells in presence or absence of 2 mM cadmium, 10 mL planktonic culture was mixed with 2 mL stop solution [water-saturated phenol 5% (pH < 7.0), ethanol 95%] (Bernstein et al., 2002; Sung et al., 2003). To discard sample processing bias, biofilm cells were suspended in a mixture of 10 mL LPTMS and 2 mL stop solution. For both samples, cells were collected by centrifugation at 1,920 × *g* for 10 min at 4°C and suspended in 700 μL of lysis buffer (sodium acetate 20 mM, EDTA 1 mM, SDS 0.5%, β-mercaptoethanol 1%, pH 5.5) and incubated 5 min at room temperature. Pre-heated acidic phenol (700 μL) was added, mixed by inversion, and incubated 10 min at room temperature. Mixtures were centrifuged at 16,000 × *g* for 5 min at 4°C. The aqueous phase was mixed with 1 volume of acid phenol:chloroform:isoamyl alcohol (25:24:1) and incubated for 5 min at room temperature. The mixture was centrifuged at 16,000 × *g* for 5 min at 4°C and the aqueous phase was mixed with 1 volume of chloroform, incubated 5 min at room temperature, and centrifuged at 16,000 × *g* for 5 min at 4°C. The resulting aqueous phase was mixed with 1 volume of isopropanol and 0.1 volume of sodium acetate 3 M (pH 5.2). The mixture was incubated overnight at -20°C. RNA was precipitated through centrifugation at 16,000 × *g* for 30 min at 4°C and washed twice with ethanol 70% (v/v). RNA samples were air dried, suspended in nuclease-free water, and treated with TURBO DNAse (AM1907) following manufacturer’s instructions. Absence of genomic DNA was controlled by PCR of the *cadA* gene with the same primers used for qPCR (Supplementary Table S2). Reverse transcription reactions were carried out using 500 ng of RNA with the ImProm II Reverse Transcription System (Promega A3800) with random primers, following manufacturer’s instructions.

### Quantitative Polymerase Chain Reaction of cDNA

Quantitative PCR was carried out with the Fast SYBRGreen Master Mix (Applied Biosystems, 4385612) in a qPCR Step One Machine (Applied Biosystems, 271003314). Primers (Supplementary Table S2) were used at 0.5 μM each. PCR conditions included a hot start step at 95°C for 20 s, followed by 40 cycles of two steps amplification (95°C for 3 s, 60°C for 30 s). For each condition, three independent experiments each with at least two technical replicates were conducted. Bacteria incubated in absence of cadmium were used as control. *Ct* values were normalized to expression of housekeeping *rpoZ* and *gyrB* genes as described (Hellemans et al., 2007). Fold changes to the control condition were assessed by one sample *t*-test. Fold changes outside of [+1, -1] range and with *P*-values below 0.05 were reported as significant.

### Construction of Recombinant *C. metallidurans* and *P. aeruginosa* Strains

Cloning procedures were performed according to standard protocols provided by the manufacturers. Primer sequences for cloning are shown in Supplementary Table S2. To characterize the function of the *urf2* gene product, the *urf2.2* gene (*RMET_RS31035*) was overexpressed in *C. metallidurans* and *P. aeruginosa*. The *urf2.2* gene was specifically amplified based on its 3′-end sequence that is absent in the *urf2.1* gene. The *urf2.2* gene was amplified from CH34 genomic DNA using an *Eco*RI restriction site flanked forward primer and a *Hind*III site flanked reverse primer. This amplicon was cloned into pGEM-T vector (Promega A1360) using T4 ligase. The recombinant vector was introduced into *E. coli* DH5α by electroporation. The *urf2.2* insert recovered from recombinant plasmid by *Eco*RI/*Hind*III digestion was ligated into the *Eco*RI/*Hind*III digested pJBTc19 vector. Thereafter, the recombinant plasmid pJB*urf2.2* was introduced into *E. coli* SL17.1 λ *pir* by electroporation. The transfer of plasmid pJB*urf2.2* into *C. metallidurans* CH34 was achieved by conjugation, where cell suspensions of donor and recipient strains were concentrated through vacuum filtration over a 0.2-μm polycarbonate filter and incubated in nutrient agar (beef extract 1 g L^-1^, yeast extract 2 g L^-1^, peptone 5 g L^-1^, and NaCl 5 g L^-1^) for 4 days at 30°C. Cells grown on the filter were spread on LPTMS agar supplemented with ampicillin 100 μg mL^-1^, tetracycline 15 μg mL^-1^, and kanamycin 1 mg mL^-1^. *P. aeruginosa* was transformed by electroporation and selection on LB agar supplemented with ampicillin 100 μg mL^-1^ and tetracycline 15 μg mL^-1^. Transformations were confirmed by double *Eco*RI/*Hind*III digestion of plasmid extraction product.

## Results

### Cadmium Exerts Inhibition of Biofilm Formation Through Decay in c-di-GMP Levels

**Figure 1:**
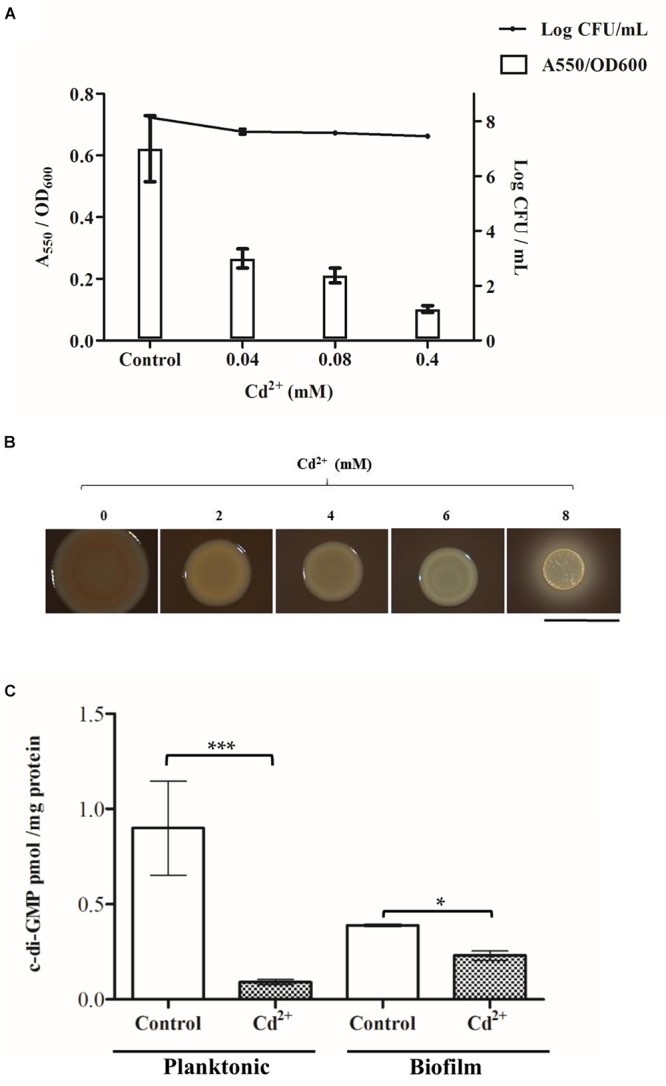
Effect of cadmium on *C. metallidurans* CH34 biofilm formation and c-di-GMP levels. **(A)** Cell attachment and cell viability after 15 h of incubation in LPTMS broth at 30°C. The means of three independent experiments and standard deviations are shown. **(B)** Macrocolonies grown on Congo Red LB agar plates at different cadmium concentrations. Images were taken after 5 days of incubation at room temperature. The red staining intensity of the macrocolony indicates the presence of EPS. The bar represents 1 cm. **(C)** c-di-GMP levels after 2 mM cadmium exposure during 45 min in LPTMS broth (planktonic) or on agar (biofilm). The c-di-GMP concentration was normalized with respect to total protein content in each sample. The means of three independent experiments and standard deviations are shown. Significant differences assessed by *t*-test: ^∗^*P* < 0.05, ^∗∗^*P* < 0.01, ^∗∗∗^*P* < 0.001.

Cell attachment assays showed that cadmium exerts a dose-dependent inhibition of early stages of biofilm formation in *C. metallidurans* CH34 (Figure 1A). Cell viability (as shown by CFU/mL) was not affected by the cadmium concentration range used, thus discarding a bias due to cell death (Figure 1A). At increasing cadmium concentration, *C. metallidurans* macrocolonies on plates showed decreasing levels of EPS that was observed by a reduction in Congo Red staining intensity (Figure 1B). A colorless extracellular matrix was observed at 8 mM cadmium. To assess the role of the c-di-GMP pathway in these phenotypes, nucleotide extracts were obtained from planktonic and biofilm cells after incubation in absence or presence of 2 mM cadmium during 45 min. The cadmium concentration 2 mM was chosen, since that concentration corresponded to the half of the MIC of cadmium for *C. metallidurans* CH34 in LPTMS broth (Table 2). In absence of cadmium, planktonic cells showed twofold higher c-di-GMP levels (∼0.8 picomol/mg protein) than biofilm cells (∼0.4 picomol/mg protein) (Figure 1C). Despite that generally higher c-di-GMP levels are expected in biofilm than in planktonic cells, it has been reported that c-di-GMP levels are dynamically modified by DGCs and PDEs in bacteria. For example, in planktonic *E. coli* cells a twofold increase of c-di-GMP levels (from 0.7 to 1.4 picomol/mg protein) in early stationary phase compared to late exponential phase has been observed (Sarenko et al., 2017). Dynamic c-di-GMP levels in biofilm cells have also been reported (Römling et al., 2013). In our study, control planktonic *C. metallidurans* cells were at post exponential phase, whereas biofilm cells were in early stage of biofilm formation. Cadmium decreased c-di-GMP levels in cells of both planktonic and biofilm lifestyles (Figure 1C). However, cadmium exerted a stronger c-di-GMP level decrease in planktonic cells, triggering an eightfold drop of c-di-GMP concentrations compared to control cells.

**Table 2:** Cadmium susceptibility parameters in *C. metallidurans* strains.

Strain	MIC (mM)	MBC (mM) (planktonic)	MBEC (mM) (biofilm)
*C. metallidurans* CH34	4	8	8
CH34 pJB3Tc19	4	8	8
CH34 pJB*urf2.2*	4	8	8
CH34 pJB*pleD^∗^*	2	2	2

The susceptibility to cadmium of *C. metallidurans* CH34 planktonic and biofilm cells was determined. For microtiter determination of antimicrobial susceptibility, the Calgary Biofilm Device was used, which allows batch culture of biofilms on peg lids (Harrison et al., 2010). Microbial biocidal endpoints were determined quantitatively using viable cell counting (CFU) following a MBEC assay (Table 2; Workentine et al., 2008; Harrison et al., 2010). Planktonic and biofilm cells showed similar tolerance levels to cadmium (Table 2).

### Searching and Selection of Potential Functional DGCs and PDEs of *C. metallidurans* CH34

Due to the potential role of c-di-GMP in the cadmium response (Figure 1C), genes encoding DGC and PDE domain-containing proteins were searched in the *C. metallidurans* CH34 genome. The functionality of proteins with GGDEF, EAL, and HD-GYP domains was predicted through analysis of the conservation of amino acid sequence motifs involved in the catalytic activity (Supplementary Figures S2–S4; Wassmann et al., 2007; Tchigvintsev et al., 2010; Lovering et al., 2011). The CH34 genome encodes 18 different GGDEF domain proteins, 10 EAL domain proteins, 12 GGDEF/EAL hybrid proteins, and 2 HD-GYP domain proteins. The functionality of each protein was predicted based on conservation of the active-site amino acids (with a maximum of one substitution), as defined by structures obtained by crystallography (Supplementary Tables S3–S5). Seventeen of 18 predicted GGDEF domain proteins showed conservation of at least six of seven amino acids that compose the active site of the GGDEF domain and, therefore, were predicted to possess DGC enzymatic activity. Nine of 10 annotated EAL domain proteins were predicted to have PDE enzymatic activity, due the conservation of at least 9 of the 10 amino acids involved in the EAL catalytic motif. In contrast, the *RMET_RS19345* gene product was predicted to be non-active.

Among the 12 hybrid proteins, 9 GGDEF domains were predicted to be active, whereas *RMET_RS19120*, *RMET_RS24490*, and *RMET_RS29670* gene products were predicted to be non-active (Supplementary Table S5). In addition, 10 EAL domains of hybrid proteins were predicted to be active, whereas *RMET_RS24490* and *RMET_RS29670* gene products were predicted to be non-active. Finally, the two loci encoding HD-GYP domain PDEs (*RMET_RS09120* and *RMET_RS20080*) were predicted to be non-active and were not further analyzed (Supplementary Figure S5).

In summary, 36 genes coding for proteins of the c-di-GMP metabolism of *C. metallidurans* CH34 were predicted to be active.

**Figure 2:**
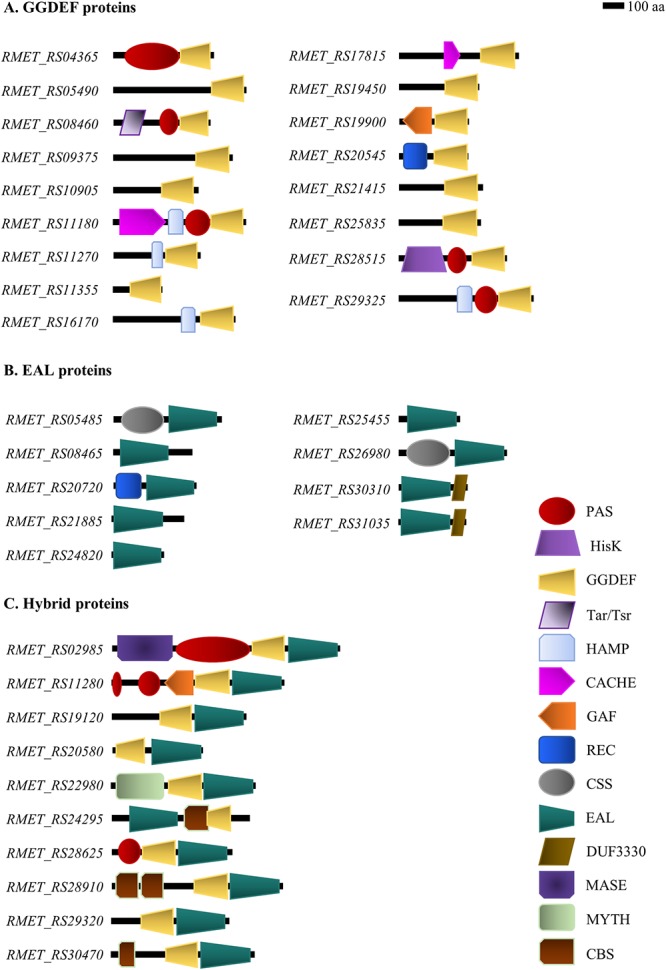
Domain-level depiction of c-di-GMP metabolism proteins from *C. metallidurans* CH34. Proteins with GGDEF/EAL domains that are catalytically active according to bioinformatic analyses are shown. **(A)** Diguanylate cyclases. **(B)** Phosphodiesterases. **(C)** Hybrid GGDEF/EAL proteins.

An accessory domain analysis was performed with the proteins predicted to possess catalytically active c-di-GMP domains (Figure 2 and Supplementary Table S6). The cytoplasmic HAMP domain, important for receptor signal(s) transduction (Matamouros et al., 2015), and the extracellular CACHE sensor domains (Upadhyay et al., 2016) were encoded exclusively in DGCs. The ionic strength sensitive domain, cystathionine beta synthase (CBS), (Biemans-Oldehinkel et al., 2006) was located exclusively in three hybrid proteins, including an uncommon localization between EAL and GGDEF domains in the *RMET_RS24295* gene product. An uncharacterized DUF3330 domain was observed in carboxy termini of the EAL domain PDE proteins encoded by the *RMET_RS30310* and *RMET_RS31035* genes (Figure 2).

In DGC proteins, a Tar/Tsr chemoreceptor domain (Tajima et al., 2011) was observed in the *RMET_RS08460* gene product, a nucleotide binding GAF domain (Heikaus et al., 2009) in the *RMET_RS19900* gene product, and a histidine kinase domain HisK in the *RMET_RS28515* gene product. The oxidative stress sensitive domain CSS was identified in the EAL domain *RMET_RS05485* and *RMET_RS26980* gene products (Huang et al., 2013). On the other hand, an integral membrane domain associated to gaseous ligand sensing (MHYT) (Galperin et al., 2005) was identified in the hybrid protein *RMET_RS22980*, and a membrane-anchoring domain MASE1 (Nikolskaya et al., 2003) in the hybrid protein *RMET_RS02985*.

Other accessory domains were transversally distributed between different proteins of the c-di-GMP metabolism. For example, the phosphorylation receiver domains (REC) were identified in DGC *RMET_RS20545* and in PDE *RMET_RS20720* gene products. The gaseous ligand binding domain PAS (Henry and Crosson, 2011) was observed in five DGCs (*RMET_RS04365*, *RMET_RS08460*, *RMET_RS11180*, *RMET_RS28515*, and *RMET_RS29325*) and three hybrid proteins (*RMET_RS02985*, *RMET_RS11280*, and *RMET_RS28625*). The domain organization analyses revealed six stand-alone GGDEF, three stand-alone EAL, and three stand-alone GGDEF/EAL domains (Figure 2 and Supplementary Table S6).

A gene orthology analysis based on comparison with the genomes of *E. coli* and *P. aeruginosa* model bacteria was performed. Based on sequence similarity and architectural domain profiles, nine CH34 orthologous DGC genes were identified in *E. coli* and *P. aeruginosa* (Supplementary Table S6). Three CH34 DGCs (*RMET_RS09375*, *RMET_RS11270*, and *RMET_RS19900*) showed a high percentage of identical residues with YedQ (27%) (Sanchez-Torres et al., 2011), TpbB (55%) (Xu et al., 2015), and YeaP (41%) (Ryjenkov et al., 2005), respectively. All aforementioned gene products are enzymes that promote EPS production and surface adherence toward robust biofilms. The analyses of the PDEs revealed that the CH34 *RMET_RS05485* and *RMET_RS26980* gene products possess high percentage of identical residues (33–34%) with the PDE YjcC from *E. coli.* It has been reported that YjcC is involved in the oxidative stress response and motility in *K. pneumoniae* (Huang et al., 2013). Similarly, the *RMET_RS20720* gene product has 30% of identical residues with RocR, which is involved in biofilm formation and virulence gene expression in *P. aeruginosa* (Rao et al., 2008). Finally, the hybrid *RMET_RS22980* gene product has 65% of identical residues with the bifunctional enzyme MucR, which is involved in biofilm dispersion in *P. aeruginosa* (Li et al., 2013).

The genomic context of CH34 c-di-GMP pathway genes and genes related to heavy metal resistance, motility, and biofilm formation was analyzed. Five c-di-GMP pathway genes were located in plasmids associated to heavy metal resistance. The *RMET_RS30470* gene that encodes a bifunctional DGC/PDE was located in a genomic island *cop-sil-nre-ncc* that is tagged as not functional (Monsieurs et al., 2011), which codes for the heavy metal resistance machinery in pMOL30. In addition, the *RMET_RS29670* gene that codes a predicted non-active protein is located in plasmid pMOL30, but outside of the heavy metal resistance genomic islands described in this replicon (Monsieurs et al., 2011). The PDE encoding *RMET_RS30310* gene is located in the pMOL30 Tn*4380 mer* operon between the *merE* gene and a gene encoding a recombinase, overlapping their ORFs in 94 bases (Supplementary Figure S5). An identical context shows the PDE encoding *RMET_RS31035* gene in the pMOL28 Tn*4378 mer* operon. Both EAL domain PDEs showed 95% sequence identity and possess the DUF3330 domain at the carboxy terminus. Both amino acidic sequences differ mainly at the carboxy terminus but conserve the domain architecture. The *RMET_RS30310* and *RMET_RS31035* genes are annotated as *urf2* (for unknown related function) genes.

Some c-di-GMP pathway genes identified are associated with biofilm formation. The *RMET_RS11355* gene is located downstream to an operon gene organization that is associated with cellulose synthesis (Supplementary Figure S5). The *RMET_RS20545* gene is located downstream to a gene cluster associated to chemotaxis response, and the *RMET_RS25455* gene is located upstream to a fimbrial synthesis gene cluster (Supplementary Figure S5).

### Search and Analyses of c-di-GMP Effectors of *C. metallidurans* CH34

*Cupriavidus metallidurans* CH34 possesses four genes (*RMET_RS05745*, *RMET_RS08705*, *RMET_RS09140*, and *RMET_RS11325*) encoding PilZ domain proteins. The PilZ domain protein encoded by the *RMET_RS09140* gene was discarded as c-di-GMP effector due to the absence of a c-di-GMP binding motif (Supplementary Figure S6). The hybrid GGDEF/EAL domain protein encoded by the *RMET_RS29670* gene, previously predicted to be catalytically non-active, was included as potential effector of a c-di-GMP pathway in *C. metallidurans* CH34 (Supplementary Table S5). Local and pairwise sequence alignments determined the CH34 genes encoding orthologous proteins of the c-di-GMP effectors Bcam1349 (*RMET_RS18290*), a transcription factor that regulates biofilm formation in *B. cenocepacia* (Fazli et al., 2014; Supplementary Figure S7) and PelD (*RMET_RS RS21490*), a degenerate DGC that regulates cationic exopolysaccharide synthesis in *P. aeruginosa* (Supplementary Figure S8).

### Effects of Cadmium on the Expression of Genes Coding for PDEs, DGCs, and Heavy Metal Resistance Proteins

**Figure 3:**
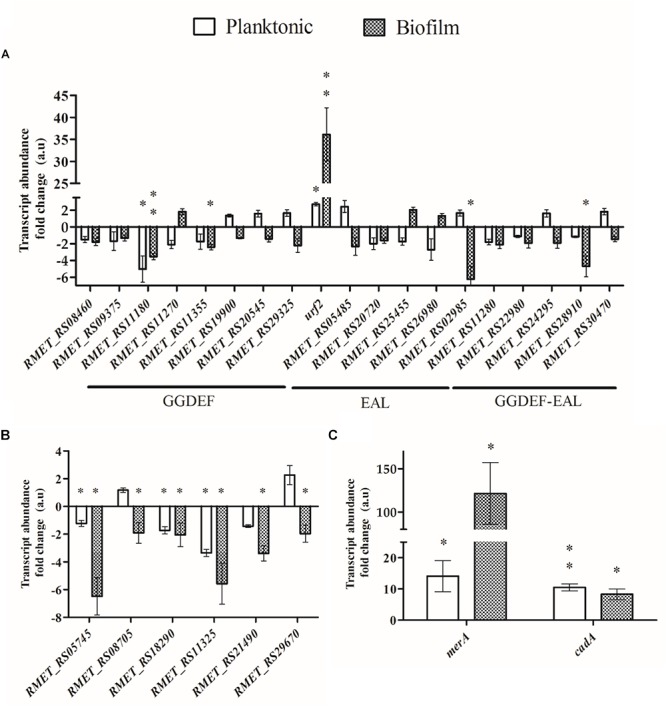
Effect of cadmium on the transcription of c-di-GMP metabolic and heavy metal resistance genes in planktonic and biofilm *C. metallidurans* CH34 cells. The transcription was measured after exposure to cadmium (2 mM) for 45 min. **(A)** Genes involved in c-di-GMP metabolism. The term *urf2* represents both *urf2.1* and *urf2.2* genes. **(B)** Genes encoding c-di-GMP effectors. **(C)** Mercury and cadmium resistance genes. Each bar represents the mean of values of three independent experiments, each analyzed in duplicate, and standard deviations. Significant differences in expression to the control condition in absence of cadmium, assessed by *t*-test, are shown in asterisk codes: ^∗^*P* < 0.05, ^∗∗^*P* < 0.01.

A subset of 19 genes coding DGCs and PDEs were selected based on three criteria: (i) genes coding for accessory domains associated to extracellular signals sensing, (ii) genomic context associated to biofilm, motility, or heavy metal resistance, and (iii) orthology with *E. coli* or *P. aeruginosa* genes that encode enzymes of the c-di-GMP metabolism. Thus, the transcription levels of these 19 genes (eight DGCs, five PDEs, and six hybrids) were quantified after 45 min exposure to 2 mM cadmium in both biofilm and planktonic cells (Figure 3A). In addition, six transcripts of c-di-GMP effectors were also quantified: three transcripts encoding PilZ domain proteins that conserve the “c-di-GMP switch” binding motif (*RMET_RS05745*, *RMET_RS08705*, and *RMET_RS11325*), one transcript encoding a degenerate hybrid GGDEF/EAL domain protein (*RMET_RS29670*), one transcript encoding for a PelD-like c-di-GMP effector (*RMET_RS21490*), and one transcript (*RMET_RS18290*) that encodes a protein with a high percentage of identical residues (53%) to Bcam1349 Figure 3B).

In both lifestyles, most of the selected transcripts (DGCs, PDEs, and hybrids) showed a non-significant abundance fold change in response to cadmium (Figure 3A). In planktonic cells, the expression of two genes changed significantly in response to cadmium. The *urf2* genes expression was upregulated (approximately threefold). Primers amplified the two *urf2* genes transcripts, hence this value is the sum of both *urf2.1* and *urf2.2* transcripts. The *RMET_RS11180* gene that encodes a DGC that possesses HAMP and CACHE extracellular signal sensing domains was strongly downregulated (approximately fivefold). In biofilm cells, the transcription of *urf2* and *RMET_RS11180* genes also changed significantly in response to cadmium. The *urf2* gene transcription increased very strongly (∼36-fold) in biofilm cells (Figure 3A). The *RMET_RS11180* gene transcription was also significantly downregulated (approximately threefold). In addition, the transcription of two hybrids proteins in biofilm cells was significantly downregulated by cadmium: *RMET_RS02985* (approximately sixfold) that encodes an integral membrane protein with a MASE1 domain, and *RMET_RS28910* (approximately fourfold) that encodes a protein with two CBS domains.

Almost all effector transcripts assessed were significantly downregulated after cadmium exposure, in higher magnitude in biofilm cells (Figure 3B). Only the *RMET_RS08705* and *RMET_RS29670* transcripts were non-significantly regulated in planktonic conditions.

In addition, the transcription of two heavy metal resistance genes was analyzed. The transcription of the *cadA* gene that encodes a Cd^2+^/ATPase protein transporter involved in cadmium resistance was monitored. As expected, after exposure to cadmium the *cadA* gene was strongly upregulated in both lifestyles at similar levels (∼10-fold) (Figure 3C). The effect of cadmium on the expression of the *merA* genes that encodes a mercuric reductase and belongs to the *mer* operons Tn*4378* and Tn*4380*, which also harbor the *urf2* genes, was also analyzed. The primers used amplify both *merA* transcripts. Interestingly, cadmium induced the transcription of the *merA* genes in both lifestyles (Figure 3C), suggesting an induction of mercury resistance by cadmium. The upregulation by cadmium of the *merA* genes was much stronger in biofilms (121-fold) than in planktonic cells (14-fold).

### The *urf2.2* Gene Encodes a Functional PDE

**Figure 4:**
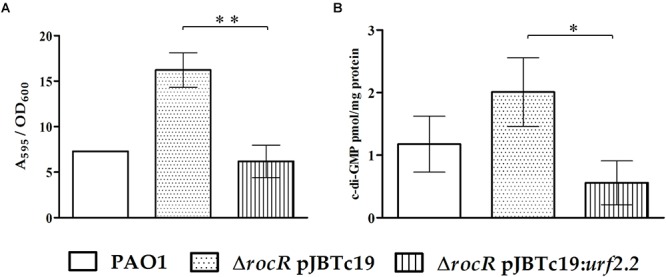
Complementation effects in *P. aeruginosa* PAO1 Δ*rocR* with pJB*urf2.2*. **(A)** Biofilm formation at 6 h of incubation in 96-well plates without cadmium. **(B)** The c-di-GMP content in planktonic cells at 6 h of incubation in Erlenmeyer flasks. The means of values of three independent experiments and standard deviations are shown. Significant differences assessed by *t*-test: ^∗^*P* < 0.05, ^∗∗^*P* < 0.01.

The *urf2.2* gene is a component of the Tn*501 mer* operon. Although Tn*501* and its derivatives were described decades ago (Brown et al., 1986), the Urf2 function remains unknown (Ei Mouali et al., 2017). Here, we are able for the first time to suggest a function. A complementation assay was developed in the PDE RocR null mutant strain *P. aeruginosa* PAO1 *ΔrocR* to evaluate the effects of the protein encoded by the *urf2.2* gene on biofilm formation and c-di-GMP levels. RocR is a PDE response regulator in the RocS1S2AR two-component signaling system, which controls bacterial biofilm formation in *P. aeruginosa* (Kuchma et al., 2005; Kulasekara et al., 2005). The complementation by the *urf2.2* gene restored biofilm formation and c-di-GMP levels (Figure 4) at levels comparable to the wild-type PAO1 strain, indicating that the *urf2.2* gene encodes a functional PDE. Based on these data we propose to rename this specific gene as *mrp2* gene for metal regulated PDE and its copy on plasmid pMOL28 as *mrp1* gene.

### Effects of PDEs on Biofilm Formation, EPS Content, and c-di-GMP Levels

**Figure 5:**
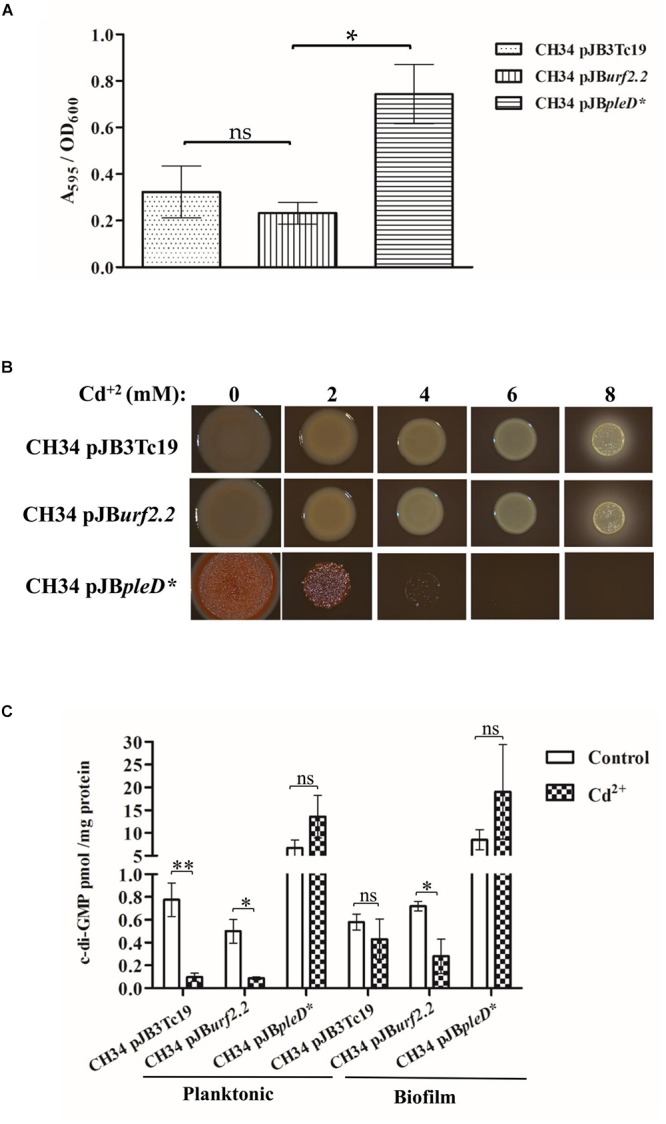
Effect of *urf2.2* overexpression and *pleD*^∗^ heterologous expression on *C. metallidurans* CH34 biofilm formation and c-di-GMP metabolism. **(A)** Biofilm formation in the absence of cadmium. The means of three independent experiments and standard deviations are shown. Significant differences assessed by *t*-test: ^∗^*P* < 0.05; ns, not significant. **(B)** Macrocolonies morphotype in the presence of cadmium. The red staining of the macrocolony indicates its EPS content. **(C)** Response in c-di-GMP levels after cadmium exposure. The means of three independent experiments and standard deviations are shown. Significant differences assessed by *t*-test: ^∗^*P* < 0.05, ^∗∗^*P* < 0.01; ns, non-significant.

The relevance of c-di-GMP levels in *C. metallidurans* CH34 for biofilm formation was also assessed through the heterologous expression of the constitutively active DGC PleD^∗^ (Pérez-Mendoza et al., 2014) and the overexpression of the *urf2.2* gene. Interestingly, the heterologous expression of the PleD^∗^ DGC increased biofilm formation in absence of cadmium (Figure 5A). In addition, cells expressing PleD^∗^ DGC increased their EPS content (Figure 5B) and c-di-GMP levels (up to >10-fold) either in absence or presence of cadmium (Figure 5C). Interestingly, in presence of cadmium c-di-GMP levels were not decreased in cells that express PleD^∗^ DGC. In contrast, the overexpression of the *urf2.2* gene has no significant effects on biofilm formation (Figure 5A) and EPS content in macrocolonies (Figure 5B). However, the overexpression of the *urf2.2* gene led to a slight decrease in c-di-GMP levels in the control planktonic cells in absence of cadmium (Figure 5C). After exposure to cadmium, c-di-GMP levels in cells that overexpress the *urf2.2* gene decreased as observed with control cells.

The effects of these alterations on cadmium susceptibility were further studied through the determination of MBC and MBEC. Interestingly, the heterologous expression of PleD^∗^ DGC resulted in significant detrimental effects on cadmium susceptibility (Table 2) in both bacterial lifestyles. Conversely, the overexpression of the *urf2.2* gene has no effect on cadmium susceptibility (Table 2).

## Discussion

This study shows that cadmium inhibits the initiation of biofilm formation in the heavy metal-resistant extremophile *C. metallidurans* CH34, which correlates with a drop in the c-di-GMP levels (Figure 1). The upregulation by cadmium of the PDE encoded by the *urf*2 genes (Figure 3A) suggests that this enzyme could be involved in the decrease in c-di-GMP concentration. In addition, the downregulation of a DGC (*RMET_RS11180*) and two hybrid proteins (*RMET_RS02985* and *RMET_RS28910*) may be involved in the second messenger decrease and should be further analyzed in future studies. We propose to rename the two *urf2* genes as *mrp* genes based on this study and the location of the two copies (*RMET_RS30310/mrp1* and *RMET_RS31035/mrp2*) within the two *mer* operons of the pMOL28 and pMOL30 plasmids. These *mer* operons are part of transposons belonging to the Tn*501*/Tn*21* family, the most widespread transposon families in terrestrial and marine environments (Brown et al., 1986; Liebert et al., 1999).

Similar to what we have observed here, a stressor reducing biofilm levels has been reported by Agulló et al. (2017) for the strain *Paraburkholderia xenovorans* LB400. This organism is a model aromatic compound-degrader, yet the toxic aromatic *p*-cymene reduced biofilm formation. This reduced biofilm formation in LB400 cells grown on *p*-cymene correlated with a decrease of the DGC protein levels that probably reduced c-di-GMP levels (Agulló et al., 2017). It has been also reported that cadmium and nickel cations inhibit biofilm formation by *Burkholderia multivorans* (Vega et al., 2014).

In our study, of the 42 proteins potentially involved in c-di-GMP metabolism that are encoded in the *C. metallidurans* CH34 genome, 36 proteins with high conservation of amino acid determinants (with a maximum of one substitution) for the catalytic activity were predicted as active, whereas 6 proteins were predicted to be non-active. The domain organization revealed a high proportion of stand-alone GGDEF (nine) and EAL (six) domains. This is in stark contrast with the 0.03 and 20% representation of these kinds of architectures within the Pfam database (Ei Mouali et al., 2017). The extremophile strain CH34 is especially well adapted to harsh niches in which heavy metals are present (Mergeay et al., 1985); therefore, the presence of unknown accessory domains in the c-di-GMP signaling pathway proteins should not be excluded.

Until this study, the role of the *urf2* gene from the mercury resistance operon was unknown (Ei Mouali et al., 2017). This *urf2* gene was the first gene encoding a protein with an EAL domain to be reported in Tn*501* (Brown et al., 1986) and Tn*21* transposon families (Liebert et al., 1999). It has been previously described that *urf2.1* (*orf2.1*) and *urf2.2* (*orf2.2*) genes are upregulated by mercury, cadmium, zinc, and lead (Monchy et al., 2007; Monsieurs et al., 2011). These heavy metals clustered into the same transcriptomic profile, i.e., regulate the same set of genes, based on microarrays data (Monsieurs et al., 2011). Our study establishes for the first time the catalytic functionality of the *urf2* (*mrp*) gene product. Its ability to functionally replace RocR in *P. aeruginosa* PAO1 Δ*rocR* in complementation assays provides the insight. RocR is a PDE response regulator in the RocS1S2AR (or SadARS) two-component signaling system in *P. aeruginosa* (Kuchma et al., 2005). This system controls bacterial biofilm formation by regulating the transcription of the *cup* fimbrial gene cluster by a poorly understood mechanism (Kulasekara et al., 2005). Structural and mechanistic studies revealed that RocR is a constitutively inhibited tetrameric PDE, which is activated after phosphorylation, a modification that exposes the catalytic EAL domains (Chen et al., 2012). This mechanism explains the absence of biofilm-related phenotype effects either by overexpression or transposon inactivation of the *roc*R gene in *P. aeruginosa* (Kuchma et al., 2005). Interestingly, the results obtained in our study showed a drop in the c-di-GMP levels and decreased biofilm formation in *P. aeruginosa* PAO1 Δ*rocR* complemented with the CH34 *mrp2* (*urf2.2*) gene (Figure 5). The discrepancy of the previous study and our work here may be explained by differences in experimental strategies used in both studies. Cells from a static culture after 8 h incubation (Kuchma et al., 2005) are in a different physiological state compared to the adhered biomass after 15 h incubation under stirred conditions (the present study). This condition generates shearing forces that promote biofilm compaction and viscosity loss in the EPS matrix (Goeres et al., 2005), which results in a biofilm that is more resistant to hydrodynamic shearing and degradation (Koechler et al., 2015). In this condition, the presence of early adherence determinants such as adhesins CupB/C could be more relevant. Adhesins expression is antagonized by RocR activity (Kulasekara et al., 2005). Thus, under the conditions used in our study, the *urf2.2* gene product could replace the RocR role in biofilm formation and c-di-GMP hydrolysis.

The absence of differences on cadmium susceptibilities (Table 2) between both bacterial lifestyles along with the significant decrease of c-di-GMP concentration in planktonic cells in response to cadmium (Figure 1C) suggest that the response exceeds planktonic lifestyle promotion and might be associated with *C. metallidurans* survival under harsh conditions and its multilayer structure to tolerate heavy metals. In support of this idea, it has been found that specific PDEs such as YjcC in *K. pneumoniae* (Huang et al., 2013), CdgR in *Salmonella typhimurium* (Hisert et al., 2005), and YgfF in *E. coli* (Lacey et al., 2010) play a role in survival during oxidative stress. Heavy metals such as cadmium destroy thiol groups in proteins, triggering an oxidative stress response in bacteria (Harrison et al., 2007; Nies, 2016). It has been proposed that the synthesis of gold nanoparticles from Au (III) ions by CH34 cells is catalyzed by sulfur-rich proteins, which are oxidized (Montero-Silva et al., 2018). Cadmium probably also oxidizes sulfur-rich proteins in CH34 cells, causing oxidative stress and inducing a specific PDE that decreases c-di-GMP concentration. The heterologous expression of a constitutively active DGC (PleD^∗^) increased the c-di-GMP levels, biofilm formation, and cadmium susceptibility in *C. metallidurans* CH34 (Figure 5). As the extracellular matrix production is an energetically costly process (Karunakaran et al., 2011), a constitutive activation of this machinery could deplete ATP cellular pools, depriving the cell to achieve efficient cadmium detoxification. By overexpression of CH34 *mrp* gene, the role of the *mrp* gene product in the c-di-GMP metabolism in response to cadmium was only observed in planktonic cells with a slight decrease in c-di-GMP that correlates with a tendency to decrease biofilm formation. More pronounced changes due to the overexpression of the *mrp* gene from the plasmid pJB*urf2.2* was not observed might be due to the high expression of the *mrp* genes from pMOL30 and pMOL28.

The *mrp* gene product domain architecture shows a C-terminal accessory domain DUF3330 (Pfam 11809), which is only present in a nitro reductase protein family. I-TASSER 3D prediction platform (Roy et al., 2010) identified five α-helix in this region beyond EAL domain toward the C-terminal region, supporting the existence of a domain in this region. Thus, the *mrp* gene product may be an exceptional case of domain organization in intracellular signaling proteins, combining a cytoplasmic sensor domain in its N-terminal extreme with a C-terminal output domain (Galperin, 2004). The DUF3330 domain may play a role in the c-di-GMP metabolism in *C. metallidurans* CH34. The *mrp2* gene product is encoded in mobile genetic elements, indicating their potential distribution to other bacteria during horizontal gene transfer (Ei Mouali et al., 2017).

Notably, a strong increase (121-fold) by cadmium of the *merA* gene expression in *C. metallidurans* biofilm cells was observed. A lower induction (14-fold) by cadmium of the *merA* gene expression was observed in planktonic cells. Previously, we have observed the upregulation by cadmium of the expression of the *merT* and *merP* genes in *C. metallidurans* planktonic cells (Rojas, 2011). The *mer* operon is also induced by cadmium in *Nitrosomonas europaea* (Park and Ely, 2008). Recently, the *merTPAGB* operon has been associated with the cadmium resistance in *C. metallidurans* (Millacura et al., 2018). These studies suggest a role of the *mer* operon in the protection toward cadmium. The flexible nature of the transcriptional regulator MerR allows the interaction with a variety of heavy metals such as cadmium, according to *in vitro* transcription studies (Ralston and O’Halloran, 1990), but with lower affinity than for mercury (Wang et al., 2016). Cadmium and mercury possess similar outer shell electron configuration (d^10^s^2^) and atomic radius (ca. 150 pm). Therefore, it will be of interest to study the effects of mercury on biofilm formation and c-di-GMP metabolism in strain CH34. The higher induction of the mercury resistance *merA* gene in the CH34 biofilm than in planktonic cells resembles the improved mercury resistance in a *Desulfovibrio desulfuricans* biofilm compared to planktonic cells (Lin et al., 2013). In CH34 biofilm cells, a lower induction of the *cadA* gene (∼10-fold) in biofilm as well as planktonic cells was observed, indicating a differential behavior of both *cad* and *mer* operons for the sessile biofilm lifestyle.

**Figure 6:**
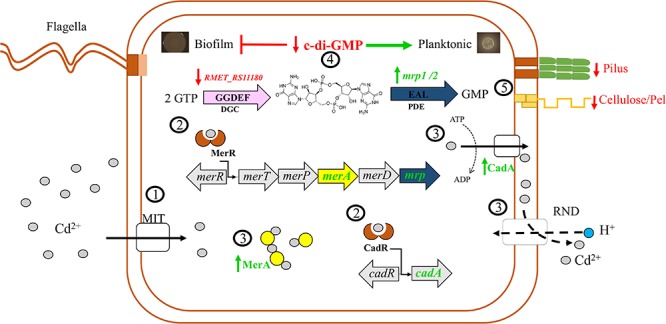
Proposed model of the *C. metallidurans* CH34 and its c-di-GMP signaling pathway in response to cadmium. **1**, Cadmium ions (gray circles) enter the cell through metal ion transporters (MIT). **2**, Cadmium induces gene expression of detoxification systems CadA, MerA, and RND antiporter pumps. Cadmium ions upregulate the phosphodiesterases (PDEs) (*mrp1* and *mrp2* genes) encoded in the mercury resistance operons Tn*4378* and Tn*4380* and downregulate the diguanylate cyclase (RMET_RS11180). **3**, MerA proteins (yellow circles) may act as chelator proteins of free cadmium cooperating with canonical detoxification systems. Efflux of cadmium is facilitated by CadA and RND antiporter pumps. **4**, Upregulation of Mrp1/2 and downregulation of RMET_RS11180 protein decreased c-di-GMP levels. **5**, The drop of c-di-GMP levels inhibits the cell adhesion (pilus) and extracellular polysaccharide synthesis (cellulose and Pel) that may favor the planktonic lifestyle. Vertical arrows stand for upregulation (green) and downregulation (red) of molecules.

Figure 6 illustrates the proposed signaling network activated by cadmium in *C. metallidurans* CH34. Cadmium cations enter the cell through metal ion transporters (MITs). In the cell, cadmium induces gene expression of the detoxification systems CadA, MerA, and RND antiporter pumps. In addition, cadmium ions upregulate the PDEs (*mrp1* and *mrp2* genes) encoded in the mercury resistance operons Tn*4378* and Tn*4380* and downregulate the DGC (RMET_RS11180). MerA proteins may act as chelator proteins of free cadmium cooperating with canonical detoxification systems. Efflux of cadmium is facilitated by CadA and RND antiporter pumps. The upregulation of PDE Mrp1/2 and downregulation of DGC RMET_ RS11180 protein decreased c-di-GMP levels. The drop of c-di-GMP levels inhibits the cell adhesion (pilus) and extracellular polysaccharide synthesis (cellulose and Pel) that may favor the planktonic lifestyle.

## Conclusion

We studied the cadmium response on the extremophile *C. metallidurans* CH34, isolated from a heavy metal-polluted slurry. For this strain cadmium inhibits the initiation of biofilm formation. A decrease in c-di-GMP levels was observed in both planktonic and biofilm cells in response to cadmium. This response to cadmium suggests that the inhibition of the biofilm formation is mediated by a decrease in c-di-GMP levels. Thus, bacterial fitness under cadmium stress favors the planktonic lifestyle. However, both lifestyles showed similar tolerance toward cadmium, suggesting the adaptive advantage of planktonic cells is to provide a means to move toward a less toxic environment. Transcription analysis suggests an active role of a previously unknown catalytic functional PDE encoded by the *urf2* genes of Tn*4378* (pMOL28) and Tn*4380* (pMOL30) *mer* operons from *C. metallidurans* CH34. Since this is the first report on the function of the *urf2* gene, the gene was renamed as *mrp2*, for “*m*etal regulated PDE.” In conclusion, this study indicates that cadmium inhibits the initiation of biofilm formation in *C. metallidurans* CH34 and that the response to cadmium involves a decrease in c-di-GMP levels and a novel metal regulated PDE.

## Author Contributions

PA-G, MS, NG, and RT conceived and designed the experiments. PA-G performed the experiments. PA-G, MS, NG, RT, SF-A, and LR analyzed the data. MS, NG, and RT contributed reagents, materials, and analysis tools. PA-G, MS, NG, RT, and SF-A wrote the manuscript.

## Conflict of Interest Statement

The authors declare that the research was conducted in the absence of any commercial or financial relationships that could be construed as a potential conflict of interest.

## Footnotes

1 http://www.jalview.org
