# Corrigendum: The Response of *Cupriavidus metallidurans* CH34 to Cadmium Involves Inhibition of the Initiation of Biofilm Formation, Decrease in Intracellular c-di-GMP Levels, and a Novel Metal Regulated Phosphodiesterase

**DOI:** 10.3389/fmicb.2019.02014

**Published:** 2019-08-30

**Authors:** Pablo Alviz-Gazitua, Sebastián Fuentes-Alburquenque, Luis A. Rojas, Raymond J. Turner, Nicolas Guiliani, Michael Seeger

**Affiliations:** ^1^Laboratorio de Comunicación Bacteriana, Departamento de Biología, Facultad de Ciencias, Universidad de Chile, Santiago, Chile; ^2^Laboratorio de Microbiología Molecular y Biotecnología Ambiental, Departamento de Química and Centro de Biotecnología, Universidad Técnica Federico Santa María, Valparaíso, Chile; ^3^Ph.D. Program of Microbiology, Facultad de Ciencias, Universidad de Chile, Santiago, Chile; ^4^Microbial Ecology of Extreme Systems Laboratory, Facultad de Ciencias Biológicas, Pontificia Universidad Católica de Chile, Santiago, Chile; ^5^Department of Chemistry, Universidad Católica del Norte, Antofagasta, Chile; ^6^Biofilm Research Group, Department of Biological Sciences, University of Calgary, Calgary, AB, Canada

**Keywords:** c-di-GMP, *Cupriavidus metallidurans*, cadmium, phosphodiesterase, biofilm, *urf2* gene, *mer* gene, PleD

In the original article, there were mistakes in the affiliations number **3**, **4**, and **5**.

Instead of “3 Ph.D. Program of Microbiology, Faculty of Sciences, University of Chile, Santiago, Chile,” it should be “3 Ph.D. Program of Microbiology, Facultad de Ciencias, Universidad de Chile, Santiago, Chile.”

Instead of “4 Microbial Ecology of Extreme Systems Laboratory, Biological Sciences Faculty, Pontifical Catholic University of Chile, Santiago, Chile,” it should be “4 Microbial Ecology of Extreme Systems Laboratory, Facultad de Ciencias Biológicas, Pontificia Universidad Católica de Chile, Santiago, Chile.”

Instead of “5 Department of Chemistry, Universidad Catoìlica del Norte, Antofagasta, Chile,” it should be “5 Department of Chemistry, Universidad Católica del Norte, Antofagasta, Chile.”

The authors apologize for these mistakes and state that corrections does not change the scientific conclusions of the article in any way. The original article has been updated.

