# Supplementary material for: The Response of Cupriavidus metallidurans CH34 to Cadmium Involves Inhibition of the Initiation of Biofilm Formation, Decrease in Intracellular c-di-GMP Levels, and a Novel Metal Regulated Phosphodiesterase
Source: Front Microbiol. 2019 Jul 9;10:1499. doi: 10.3389/fmicb.2019.01499 (PMC6629876; doi:10.3389/fmicb.2019.01499)
Supplement: Supplementary file 1 [file Table_1.DOCX]

**Table S1. New and old locus tags correspondence in the *C. metallidurans* CH34 genome**

| **DGCs with GGDEF domain** | |
| --- | --- |
| **New locus tag** | **Old locus tag** |
| *RMET_RS04365* | *Rmet_0867* |
| *RMET_RS05490* | *Rmet_1091* |
| *RMET_RS08460* | *Rmet_1688* |
| *RMET_RS09375* | *Rmet_1872* |
| *RMET_RS10905* | *Rmet_2173* |
| *RMET_RS11180* | *Rmet_2227* |
| *RMET_RS11270* | *Rmet_2245* |
| *RMET_RS11355* | *Rmet_2262* |
| *RMET_RS16170* | *Rmet_3225* |
| *RMET_RS17815* | *Rmet_3547* |
| *RMET_RS19450* | *Rmet_3745* |
| *RMET_RS19900* | *Rmet_3840* |
| *RMET_RS20545* | *Rmet_3967* |
| *RMET_RS21415* | *Rmet_4143* |
| *RMET_RS25835* | *Rmet_5041* |
| *RMET_RS28515* | *Rmet_5581* |
| *RMET_RS28900* | *Rmet_5658* |
| *RMET_RS29325* | *Rmet_5751* |
| **PDEs with EAL domain** | |
| **New locus tag** | **Old locus tag** |
| *RMET_RS05485* | *Rmet_1090* |
| *RMET_RS08465* | *Rmet_1689* |
| *RMET_RS19345* | *Rmet_3724* |

*(Table S1)*

| *RMET_RS20720* | *Rmet_4002* |
| --- | --- |
| *RMET_RS21885* | *Rmet_4237* |
| *RMET_RS24820* | *Rmet_4836* |
| *RMET_RS25455* | *Rmet_4962* |
| *RMET_RS26980* | *Rmet_5276* |
| *RMET_RS30310* | *Rmet_6177* |
| *RMET_RS31035* | *Rmet_6186* |
| **Hybrid Proteins** | |
| **New locus tag** | **Old locus tag** |
| *RMET_RS02985* | *Rmet_0591* |
| *RMET_RS11280* | *Rmet_2247* |
| *RMET_RS19120* | *Rmet_3679* |
| *RMET_RS20580* | *Rmet_3974* |
| *RMET_RS24490* | *Rmet_4766* |
| *RMET_RS22980* | *Rmet_4460* |
| *RMET_RS24295* | *Rmet_4726* |
| *RMET_RS28625* | *Rmet_5603* |
| *RMET_RS28910* | *Rmet_5660* |
| *RMET_RS29320* | *Rmet_5750* |
| *RMET_RS29670* | *Rmet_6058* |
| *RMET_RS30470* | *Rmet_6140* |

**Table S2. Plasmids and strains used in this study**

| **ORF** | **Orientation** | **5´- 3' Sequence** | **Amplicon (pb)** |
| --- | --- | --- | --- |
| *RMET_RS02985* | Fwd | AGCGCGGTTTCGGTGATTT | 127 |
|  | Rev | TGGTGTCGATCAACCTGTCT |  |
| *RMET_RS05485* | Fwd | GTGCATTGCTTGGATGGCTT | 145 |
|  | Rev | ACGCCTACGCATTCGTTGT |  |
| *RMET_RS05490* | Fwd | GCTTGGGTTCGCAACTCCTT | 124 |
|  | Rev | ACATGCTGGTGCATTGTGCT |  |
| *RMET_RS08460* | Fwd | GGAATCATATGCCCAACCGCTAAG | 120 |
|  | Rev | ACGTATCCGTCAAACCGCAACA |  |
| *RMET_RS08465* | Fwd | GCCAAGAGTACCGATTGCGT | 133 |
|  | Rev | ATGTGGATGCGCTGCACTTT |  |
| *RMET_RS09375* | Fwd | GGAAACAGCGGCCTGATCAT | 135 |
|  | Rev | AATTGCCAGCGTGAAGCAGA |  |
| *RMET_RS11180* | Fwd | TGGCCATCGCCAATCATGTT | 125 |
|  | Rev | AGAAAGGCCCGTGATCTGGT |  |
| *RMET_RS11270* | Fwd | CGCGTTTGGACAGCGAATG | 113 |
|  | Rev | CGTGCTGAAGCTGGTTTTGC |  |
| *RMET_RS11280* | Fwd | TGCCGTCGCGTGGAAAGAAT | 121 |
|  | Rev | TGTCGAATGCGGCAAGCAGA |  |
| *RMET_RS11355* | Fwd | CCATCGCCGGACATCGTTT | 139 |
|  | Rev | GCCTCGCTTGGCTTCATACA |  |
| *RMET_RS16170* | Fwd | TCTGGCTGCATTCGCTGTTT | 133 |
|  | Rev | CGGATTTCGCCACGGTAGTT |  |
| *RMET_RS19900* | Fwd | GCCGACGTCACGATCGATTA | 139 |
|  | Rev | CGTTTGGGCCAGATTGCTTG |  |
| *RMET_RS20545* | Fwd | AAGCTTCCGGATTCGATCGAGTTG | 148 |
|  | Rev | TTAGCCGCTGCAGTTCGAGATT |  |
| *RMET_RS20720* | Fwd | GCTGGTCAGATCACGCAACT | 127 |
|  | Rev | TGGCCAAGCCGCTGAATTT |  |
| *RMET_RS22980* | Fwd | GTCGATGGCGGTCAACCTTT | 115 |
|  | Rev | TCGAGCGTCAATCGTCTTGG |  |

*(Table S2)*

| *RMET_RS24295* | Fwd | GCGATCGTTGAATGGCTTGA | 132 |
| --- | --- | --- | --- |
|  | Rev | TATGCGAATCCGCTGACGTT |  |
| *RMET_RS24820* | Fwd | GCGGGCGATCTTCACGTAAT | 137 |
|  | Rev | CGCTGGAATCGCTGACCAA |  |
| *RMET_RS25455* | Fwd | GGCTGATAGCAAGCCACGAA | 144 |
|  | Rev | ACCCCGCGAGTCTGGTAAAT |  |
| *RMET_RS26980* | Fwd | ACGTTGGCCACGCTGTTT | 139 |
|  | Rev | TGATTGCCTGCGTCATGACC |  |
| *RMET_RS28910* | Fwd | GTGCATGGCCAGATGGTTCA | 149 |
|  | Rev | CGAAAGAACGGCGAGCTGTA |  |
| *RMET_RS29325* | Fwd | ACGCCTTCGAAAGCCAGTT | 123 |
|  | Rev | TGGCATGGCCGTGTATGTC |  |
| *RMET_RS29670* | Fwd | TCCGCCAGGTTAAGCAAACA | 127 |
|  | Rev | AAGCGAATCGTGCCCTTTGT |  |
| *RMET_RS30470* | Fwd | TCGCGCCTTTATCCATGACA | 132 |
|  | Rev | ACCGACACTTGCCAAACGTA |  |
| *urf2* | Fwd | CACGCTCAAGATCGACCAAT | 133 |
|  | Rev | AAGACTCGCCGATGTTTCCA |  |
| *rpoZ* | Fwd | CGCGTATTACCGTCGAAGA | 123 |
|  | Rev | TGTCCTTTGCCTCGACCTT |  |
| *gyrB* | Fwd | AGAAAACGAGGTCGCCAAGA | 132 |
|  | Rev | GAAACGAGCTTGTCCTTGGT |  |
| *urf2.2* | Fwd | AATAATAAGCTTATGAGCGCTTTCCGG | 1050 |
|  | Rev | AATAATGAATTCATGCCGCCGCCGGCA |  |

| **Table S3. Prediction of the diguanylate cyclase functionality of GGDEF domain proteins encoded in *C. metallidurans* CH34 genome.** | | | | | |
| --- | --- | --- | --- | --- | --- |
| **Locus** | **Replicon** | **Active site (7 aa)** | **Ip^a^  site (3 aa)** | **Is^b^ site (1 o 2 aa)** | **Prediction** |
| ***RMET_RS04365*** | CHR1 | 6 | 0 | 0 | Active |
| ***RMET_RS05490*** | CHR1 | 7 | 3 | 0 | Active |
| ***RMET_RS08460*** | CHR1 | 6 | 1 | 0 | Active |
| ***RMET_RS09375*** | CHR1 | 7 | 3 | 2 | Active |
| ***RMET_RS10905*** | CHR1 | 7 | 3 | 0 | Active |
| ***RMET_RS11180*** | CHR1 | 6 | 2 | 0 | Active |
| ***RMET_RS11270*** | CHR1 | 6 | 0 | 0 | Active |
| ***RMET_RS11355*** | CHR1 | 7 | 0 | 0 | Active |
| ***RMET_RS16170*** | CHR1 | 6 | 3 | 0 | Active |
| ***RMET_RS17815*** | CHR1 | 7 | 1 | 2 | Active |
| ***RMET_RS19450*** | CHR2 | 6 | 0 | 0 | Active |
| ***RMET_RS19900*** | CHR2 | 6 | 1 | 2 | Active |
| ***RMET_RS20545*** | CHR2 | 7 | 3 | 2 | Active |
| ***RMET_RS21415*** | CHR2 | 7 | 3 | 0 | Active |
| ***RMET_RS25835*** | CHR2 | 7 | 0 | 1 | Active |
| ***RMET_RS28515*** | CHR2 | 7 | 0 | 0 | Active |
| ***RMET_RS28900*** | CHR2 | 3 | 0 | 0 | Non Active |
| ***RMET_RS29325*** | CHR2 | 7 | 0 | 0 | Active |

a: Primary inhibition site, b: Secondary inhibition site

| **Table S4. Prediction of the phosphodiesterase functionality of EAL domain proteins encoded in *C. metallidurans* CH34 genome.** | | | |
| --- | --- | --- | --- |
| **Locus** | **Replicon** | **Active site (10 aa)** | **Prediction** |
| ***RMET_RS05485*** | CHR1 | 10 | Active |
| ***RMET_RS08465*** | CHR1 | 9 | Active |
| ***RMET_RS19345*** | CHR2 | 6 | Non active |
| ***RMET_RS20720*** | CHR2 | 10 | Active |
| ***RMET_RS21885*** | CHR2 | 9 | Active |
| ***RMET_RS24820*** | CHR2 | 10 | Active |
| ***RMET_RS25455*** | CHR2 | 9 | Active |
| ***RMET_RS26980*** | CHR2 | 9 | Active |
| ***RMET_RS30310*** | pMOL30 | 10 | Active |
| ***RMET_RS31035*** | pMOL28 | 10 | Active |

| **Table S5. Bioinformatic prediction of the catalytic functionality of EAL & GGDEF domain proteins encoded in *C. metallidurans* CH34 genome.**  **Hybrid Proteins** | | | | | | | |
| --- | --- | --- | --- | --- | --- | --- | --- |
|  |  | **GGDEF** | | | | **EAL** | |
| **Locus** | **Replicon** | **Active site (7 aa)** | **Ip^a^ site (3 aa)** | **Is^b^ site (1 o 2 aa)** | **Prediction** | **Active site (10 aa)** | **Prediction** |
| ***RMET_RS02985*** | CHR1 | 7 | 2 | 0 | Active | 10 | Active |
| ***RMET_RS11280*** | CHR1 | 4 | 2 | 0 | Active | 10 | Active |
| ***RMET_RS19120*** | CHR2 | 3 | 1 | 0 | Non active | 10 | Active |
| ***RMET_RS20580*** | CHR2 | 5 | 0 | 0 | Non active | 9 | Active |
| ***RMET_RS24490*** | CHR2 | 2 | 0 | 0 | Non active | 6 | Non active |
| ***RMET_RS22980*** | CHR2 | 7 | 0 | 0 | Active | 10 | Active |
| ***RMET_RS24295*** | CHR2 | 7 | 0 | 0 | Active | 10 | Active |
| ***RMET_RS28625*** | CHR2 | 7 | 1 | 0 | Active | 10 | Active |
| ***RMET_RS28910*** | CHR2 | 6 | 1 | 2 | Active | 10 | Active |
| ***RMET_RS29320*** | CHR2 | 6 | 0 | 0 | Active | 10 | Active |
| ***RMET_RS29670*** | pMOL30 | 1 | 1 | 0 | Non active | 4 | Non active |
| ***RMET_RS30470*** | pMOL30 | 7 | 2 | 0 | Active | 10 | Active |

a: Primary inhibition site, b: Secondary inhibition site

**Table S6. Orthology analysis of putative DGC/PDEs encoded in *C. metallidurans* CH34 and in *E. coli* K12 and *P. aeruginosa* PAO1 genomes**. The length of each amino acidic sequence is indicated between brackets. Genes in bold were selected for transcriptomic analysis.

| ***C. metallidurans* CH34 gene products** | | **Orthologous candidate** | | | | |
| --- | --- | --- | --- | --- | --- | --- |
| **Locus** | **Domain architecture (aa)** | **Ref. Sequence** | **Organism** | **Function** | **% Id** | **Domain architecture (aa)** |
| **GGDEF domain coding ORFs** |  |  |  |  |  |  |
| ***RMET_RS04365*** | **PAS-GGDEF(492)** | **MorA** | ***P. aeruginosa* PAO1** | **DGC-PDE** | **73** | **BaeS-PAS-GGDEF-EAL (1415)** |
| *RMET_RS05490* | GGDEF (653) | NP_251560.1 | *P. aeruginosa* PAO1 | Hyp | 33 | GGDEF (525) |
| ***RMET_RS08460*** | **Tar_Tsr-PAS-GGDEF (546)** | **NP_249266.1** | ***P. aeruginosa* PAO1** | **Hyp** | **44** | **PBP-PAS-PAS-PAS-GGDEF-EAL (1245)** |
| ***RMET_RS09375*** | **GGDEF (586)** | **YedQ** | ***E. coli* K12** | **DGC** | **27** | **GGDEF (558)** |
| *RMET_RS10905* | GGDEF (416) | AID76695.1 | *P. aeruginosa* PAO1 | Hyp | 43 | DNAPollII- SMCN-GGDEF (671) |
| ***RMET_RS11180*** | **CACHE-HAMP-PAS-GGDEF (654)** | **EHS35231.1** | ***P. aeruginosa* PAO1** | **Hyp** | **30** | **CHASE-HAMP-PAS-GGDEF (638)** |
| ***RMET_RS11270*** | **HAMP-GGDEF (426)** | **TpbB** | ***P. aeruginosa* PAO1** | **DGC** | **55** | **HAMP-GGDEF (435)** |
| ***RMET_RS11355*** | **GGDEF (237)** | **AID76049.1** | ***E. coli* K12** | **DGC** | **42** | **REC-REC-GGDEF (542)** |
| *RMET_RS16170* | HAMP-GGDEF (599) | NP_251560.1 | *P. aeruginosa* PAO1 | Hyp | 45 | GGDEF (525) |
| *RMET_RS17815* | CACHE-GGDEF (587) | YedQ | *E. coli* K12 | DGC | 27 | GGDEF (558) |
| *RMET_RS19450* | GGDEF (392) | NP_250418.1 | *P. aeruginosa* PAO1 | Hyp | 37 | MHYT- GGDEF (685) |
| ***RMET_RS19900*** | **GAF-GGDEF (338)** | **YeaP** | ***E. coli* K12** | **DGC** | **41** | **GAF-GGDEF (341)** |
| ***RMET_RS20545*** | **REC-GGDEF (335)** | **WspR** | ***P. aeruginosa* PAO1** | **Hyp** | **72** | **REC-GGDEF (347)** |
| *RMET_RS21415* | GGDEF (409) | AID74892.1 | *P. aeruginosa* PAO1 | DGC | 44 | REC-GGDEF (347) |
| *RMET_RS25835* | GGDEF (400) | WP_077873855.1 | *E. coli* K12 | Hyp | 35 | GGDEF (252) |
| *RMET_RS28515* | HisK-PAS-GGDEF (530) | NP_252391.1 | *P. aeruginosa* PAO1 | Hyp | 35 | REC-REC-GGDEF (347) |
| ***RMET_RS29325*** | **HAMP-PAS-GGDEF (662)** | **NP_249266.1** | ***P. aeruginosa* PAO1** | **Hyp** | **30** | **PBP-PAS-PAS-PAS-GGDEF (1245)** |

*(Table S6)*

| **EAL domain coding ORFs** |  |  |  |  |  |  |
| --- | --- | --- | --- | --- | --- | --- |
| ***RMET_RS05485*** | **CSS-EAL (530)** | **YjcC** | ***E. coli* K12** | **PDE** | **33** | **CSS-EAL (516)** |
| ***RMET_RS08465*** | **EAL (385)** | **MucR** | ***P. aeruginosa* PAO1** | **PDE** | **33** | **MHYT-GGDEF-EAL (685)** |
| ***RMET_RS20720*** | **REC-EAL (404)** | **RocR** | ***P. aeruginosa* PAO1** | **PDE** | **30** | **REC-EAL (392)** |
| *RMET_RS21885* | EAL (353) | AID75563.1 | *P. aeruginosa* PAO1 | PDE | 33 | GGDEF-EAL (687) |
| ***RMET_RS24820*** | EAL (254) | AID74466.1 | *P. aeruginosa* PAO1 | Hyp | 42 | EAL-CBS-GGDEF (582) |
| ***RMET_RS25455*** | **EAL (298)** | **MorA** | ***P. aeruginosa* PAO1** | **PDE** | **36** | **HisK-PAS-GGDEF- EAL (1415)** |
| ***RMET_RS26980*** | **CSS-EAL (529)** | **YjcC** | ***E. coli* K12** | **PDE** | **34** | **CSS-EAL (516)** |
| ***RMET_RS30310*** | **EAL-DUF3330 (333)** | **MorA** | ***P. aeruginosa* PAO1** | **PDE** | **45** | **HisK-PAS-GGDEF- EAL (1415)** |
| ***RMET_RS31035*** | **EAL-DUF3330 (329)** | **MorA** | ***P. aeruginosa* PAO1** | **PDE** | **45** | **HisK-PAS-GGDEF- EAL (1415)** |
| **Hybrid proteins coding ORFs** |  |  |  |  |  |  |
| ***RMET_RS02985*** | **MASE1-PAS-GGDEF-EAL (1121)** | **NP_249872.1** | ***P. aeruginosa* PAO1** | **Hyp** | **47** | **PAS-PAS-GGDEF- EAL (1120)** |
| ***RMET_RS11280*** | **PAS-PAS-GAF-GGDEF-EAL (850)** | **DosP** | ***E. coli* K12** | **PDE** | **32** | **PAS-PAS-GGDEF - EAL (799)** |
| *RMET_RS19120* | GGDEF-EAL (660) | AID74522.1 | *P. aeruginosa* PAO1 | Hyp | 33 | MHYT-GGDEF-EAL (726) |
| *RMET_RS20580* | GGDEF-EAL (441) | AID75563.1 | *P. aeruginosa* PAO1 | Hyp | 39 | GGDEF-EAL (687) |
| ***RMET_RS22980*** | **MHYT-GGDEF-EAL (709)** | **MucR** | ***P. aeruginosa* PAO1** | **PDE** | **65** | **MHYT-GGDEF-EAL (685)** |
| ***RMET_RS24295*** | **EAL-CBS-GGDEF (676)** | **WP_079279815.1** | ***P. aeruginosa* PAO1** | **Hyp** | **34** | **EAL-CBS-GGDEF (601)** |
| *RMET_RS28625* | PAS-GGDEF-EAL (589) | MucR | *P. aeruginosa* PAO1 | PDE | 42 | MHYT-GGDEF-EAL (685) |
| ***RMET_RS28910*** | **CBS-CBS-GGDEF-EAL (839)** | **NP_249266.1** | ***P. aeruginosa* PAO1** | **PDE** | **48** | **PBP - PAS - GGDEF-EAL (1245)** |

*(Table S6)*

| *RMET_RS29320* | GGDEF-EAL (581) | AID74522.1 | *P. aeruginosa* PAO1 | Hyp | 41 | MHYT-GGDEF-EAL (726) |
| --- | --- | --- | --- | --- | --- | --- |
| ***RMET_RS30470*** | **CBS-GGDEF-EAL (705)** | **AID71820.1** | ***P. aeruginosa* PAO1** | **Hyp** | **40** | **PBP-PAS-PAS-PAS-GGDEF-EAL (1245)** |
