# Supplementary figures and images for: The Response of Cupriavidus metallidurans CH34 to Cadmium Involves Inhibition of the Initiation of Biofilm Formation, Decrease in Intracellular c-di-GMP Levels, and a Novel Metal Regulated Phosphodiesterase
Source: Front Microbiol. 2019 Jul 9;10:1499. doi: 10.3389/fmicb.2019.01499 (PMC6629876; doi:10.3389/fmicb.2019.01499)

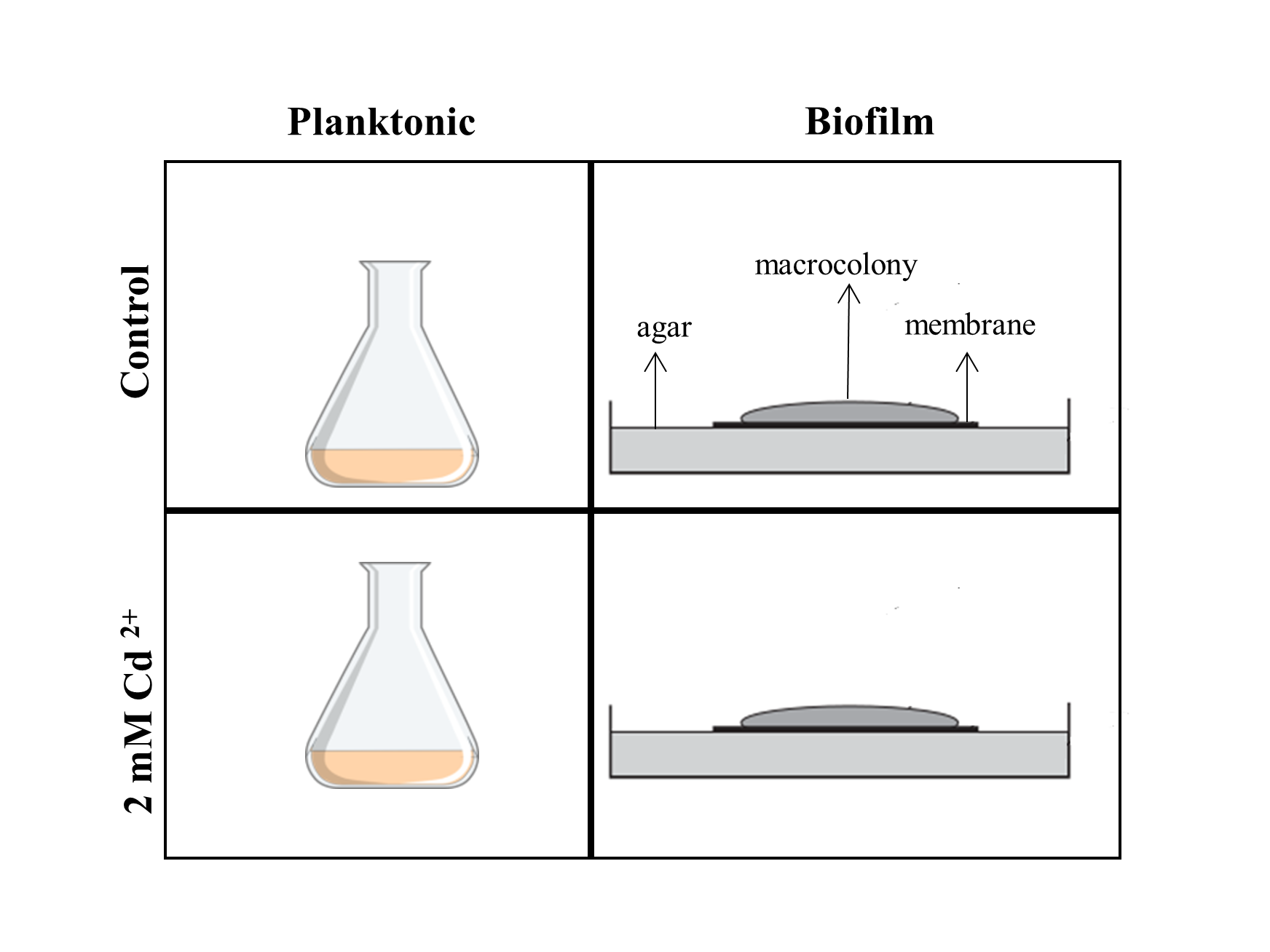

Supplement: Supplementary file 2 [file Image_1.TIF]

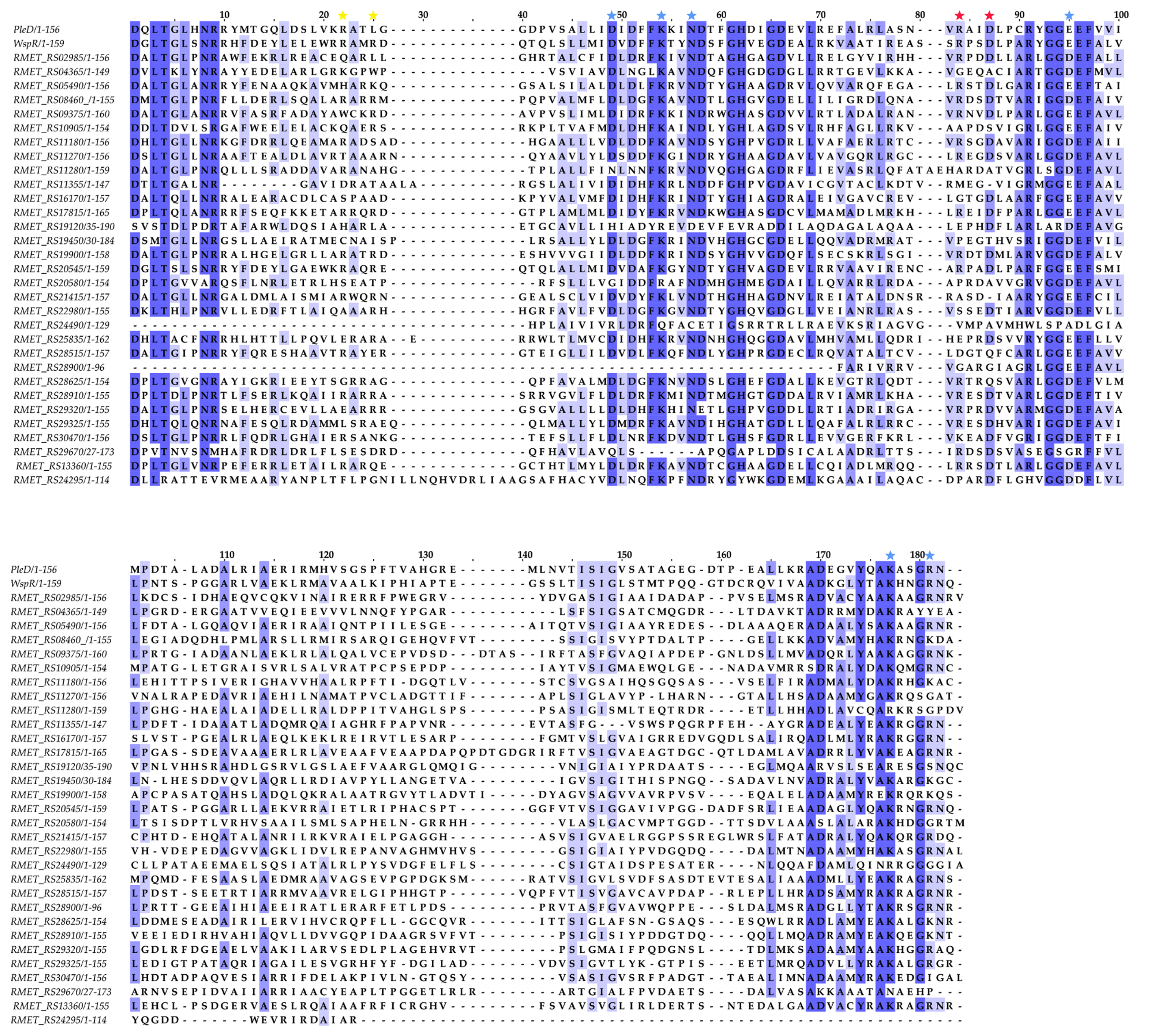

Supplement: Supplementary file 3 [file Image_2.TIF]

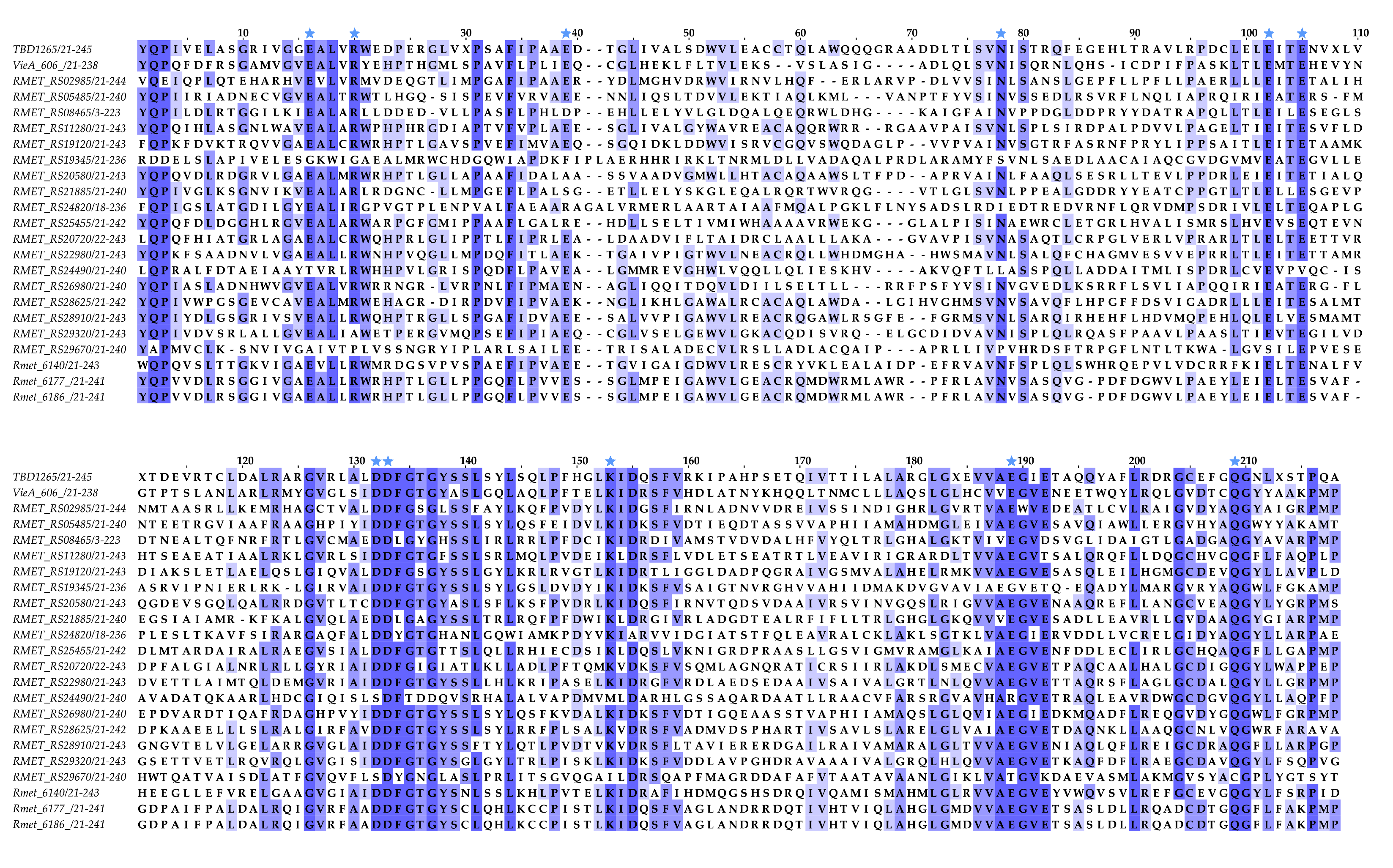

Supplement: Supplementary file 4 [file Image_3.TIF]

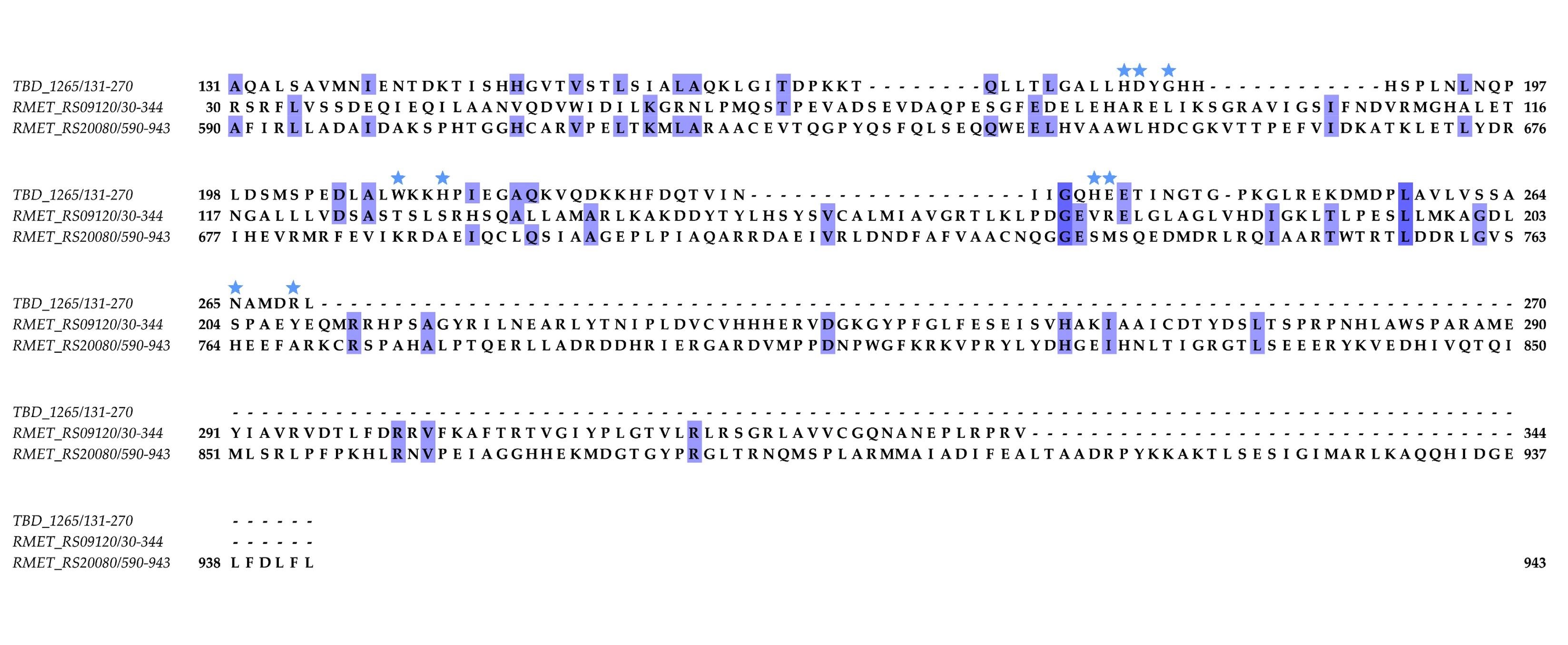

Supplement: Supplementary file 5 [file Image_4.TIF]

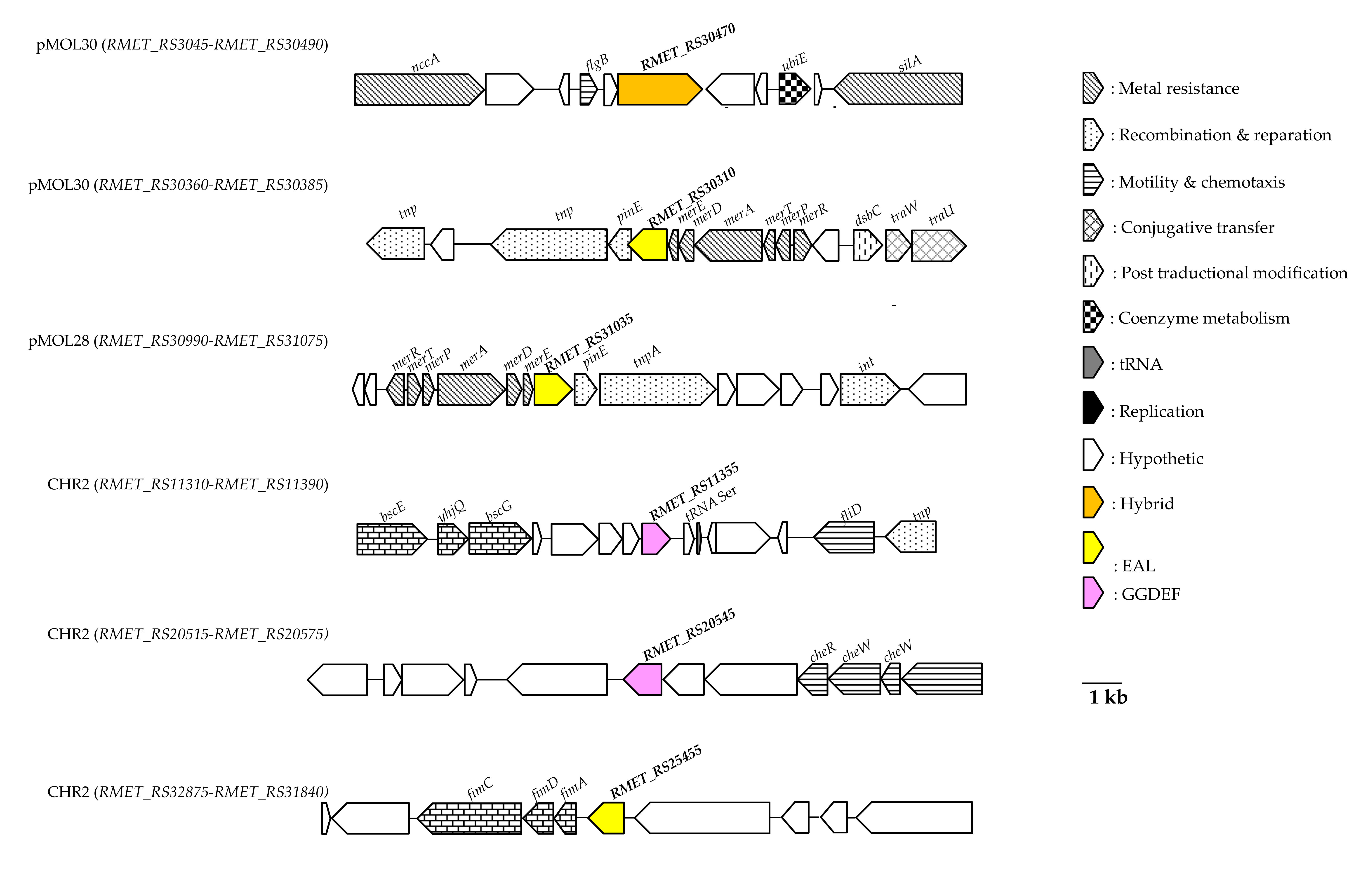

Supplement: Supplementary file 6 [file Image_5.TIFF]

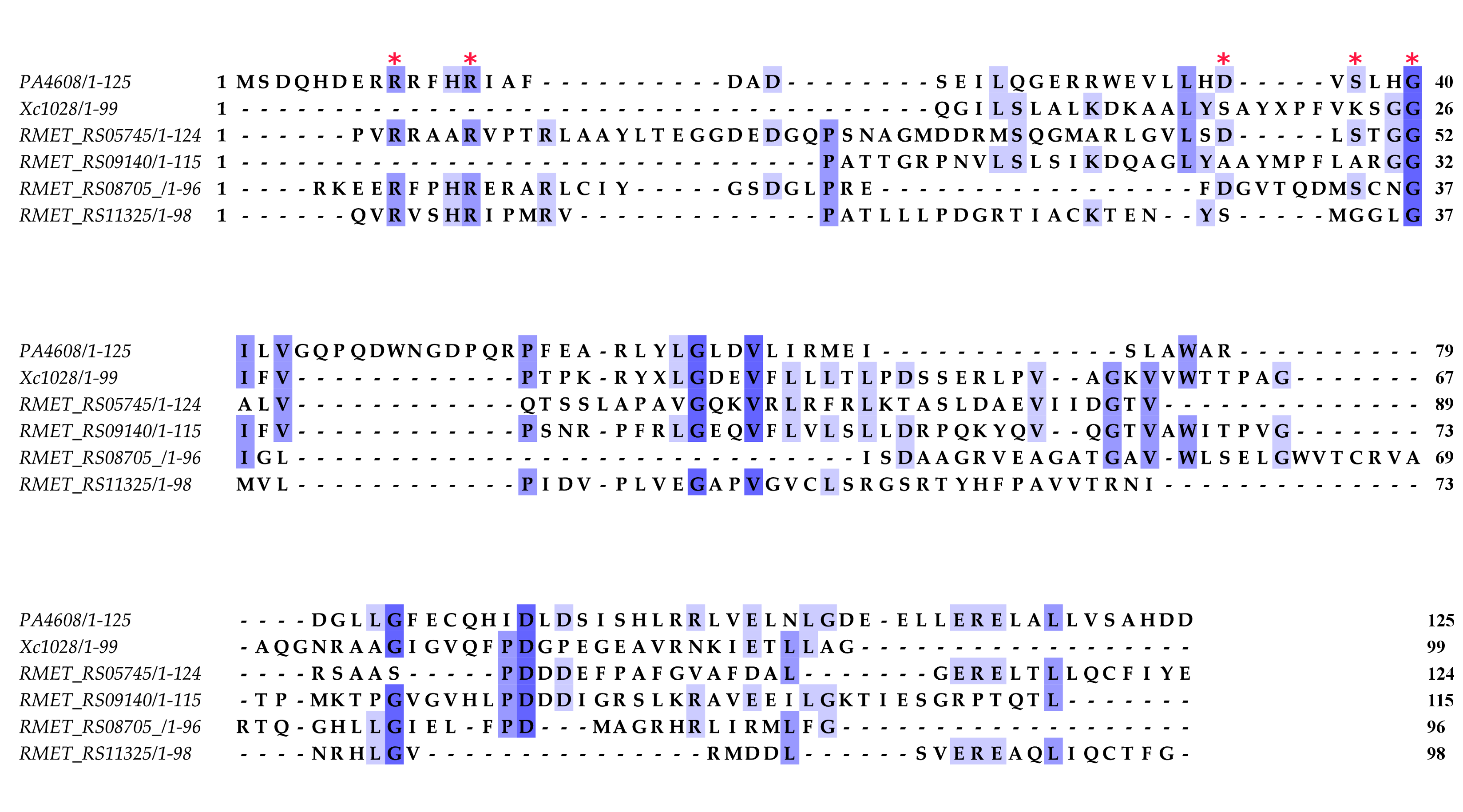

Supplement: Supplementary file 7 [file Image_6.TIF]

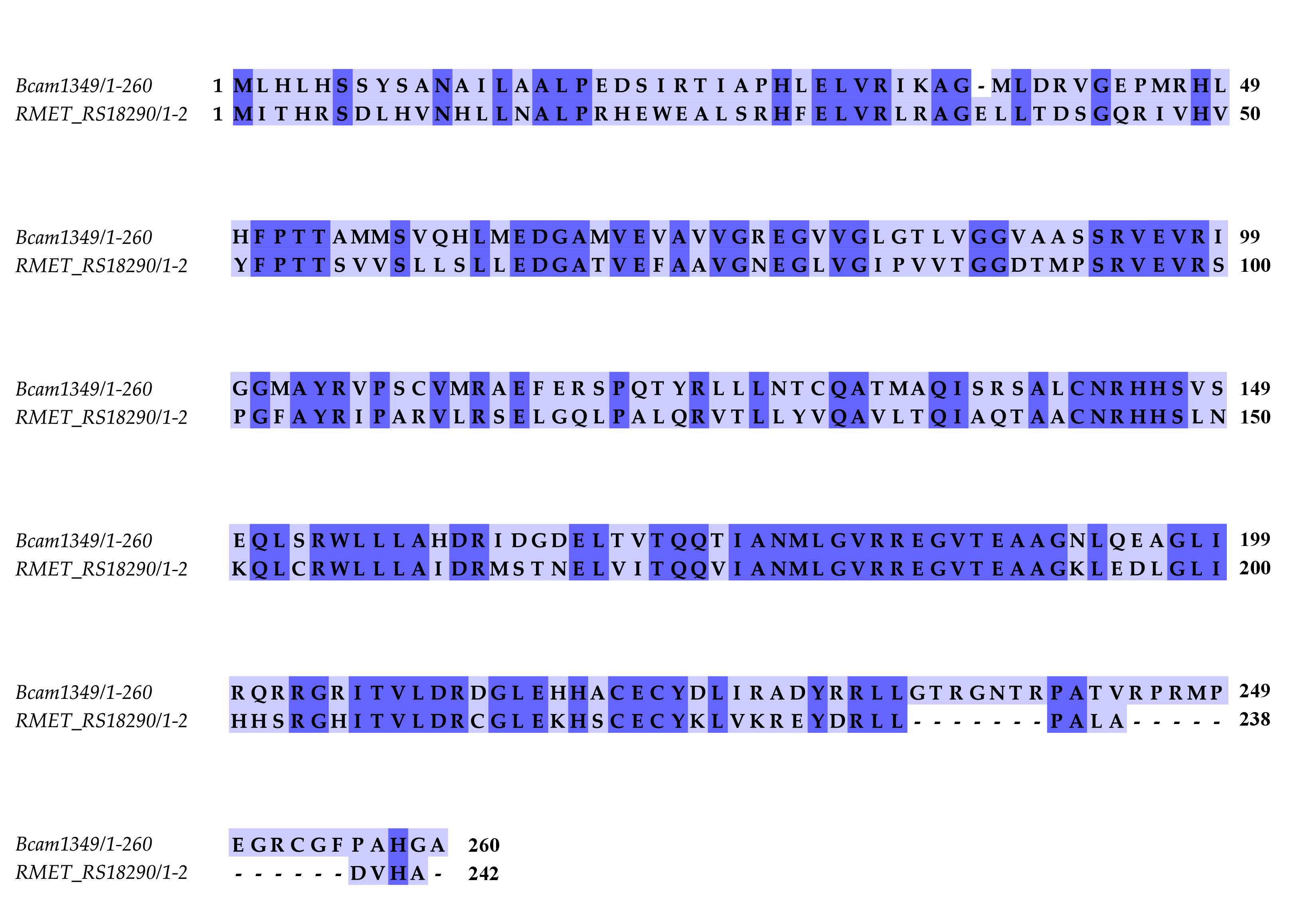

Supplement: Supplementary file 8 [file Image_7.TIF]

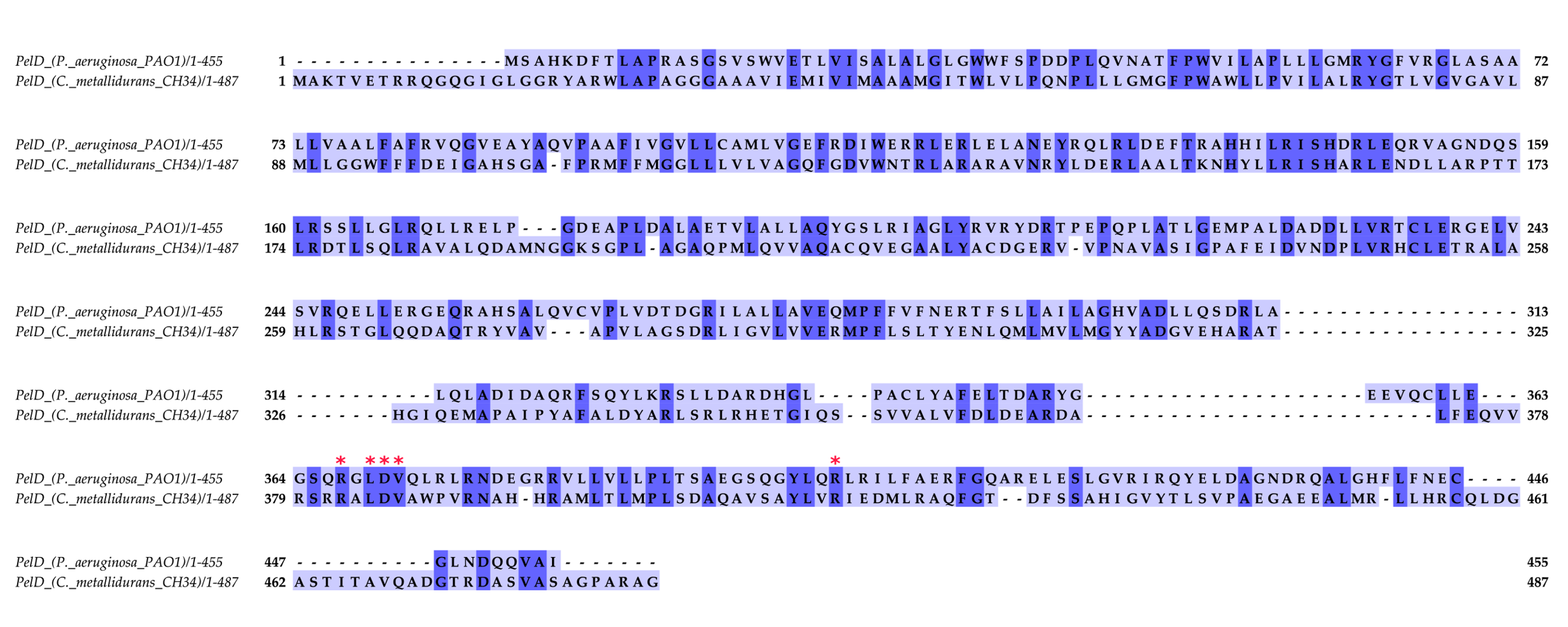

Supplement: Supplementary file 9 [file Image_8.TIF]
